# Adjustment of nursing home quality indicators

**DOI:** 10.1186/1472-6963-10-96

**Published:** 2010-04-15

**Authors:** Richard N Jones, John P Hirdes, Jeffrey W Poss, Maureen Kelly, Katharine Berg, Brant E Fries, John N Morris

**Affiliations:** 1 Institute for Aging Research, Hebrew SeniorLife, Boston MA, USA; 2 Department of Medicine, Division of Gerontology, Beth Israel Deaconess Medical Center, Harvard Medical School, USA; 3 Department of Health Studies and Gerontology, Waterloo, Ontario, Canada; 4 Homewood Research Institute, Guelph, Ontario, Canada; 5 Canadian Institute for Health Information, Ontario, Canada; 6 Department of Physical Therapy, University of Toronto, Ontario, Canada; 7 Institute of Gerontology, University of Michigan and VA Ann Arbor Healthcare Systems, Ann Arbor, MI, USA

## Abstract

**Background:**

This manuscript describes a method for adjustment of nursing home quality indicators (QIs) defined using the Center for Medicaid & Medicare Services (CMS) nursing home resident assessment system, the Minimum Data Set (MDS). QIs are intended to characterize quality of care delivered in a facility. Threats to the validity of the measurement of presumed quality of care include baseline resident health and functional status, pattern of comorbidities, and facility case mix. The goal of obtaining a valid facility-level estimate of true quality of care should include adjustment for resident- and facility-level sources of variability.

**Methods:**

We present a practical and efficient method to achieve risk adjustment using restriction and indirect and direct standardization. We present information on validity by comparing QIs estimated with the new algorithm to one currently used by CMS.

**Results:**

More than half of the new QIs achieved a "Moderate" validation level.

**Conclusions:**

Given the comprehensive approach and the positive findings to date, research using the new quality indicators is warranted to provide further evidence of their validity and utility and to encourage their use in quality improvement activities.

## Background

In 1986, the Institute of Medicine Committee on Nursing Home Regulation made recommendations to Congress [1] to improve quality of care in nursing homes (NH). One was the systematic collection of standardized data on all NH residents: a minimum data set. Under this mandate, the U.S. Center for Medicare and Medicaid Services (CMS) implemented the Resident Assessment Instrument - Minimum Data Set (MDS). All U.S. long-term care (LTC) facilities must complete standardized MDS assessments of each resident to participate.

The MDS includes a clinical assessment of over 400 items covering demographics, medical condition, cognitive, physical, emotional and social functioning, medical diagnoses, therapies, treatments and medication use. The aggregation of individual MDS assessments into archives can be used to generate representative data sets used for prospective payment systems [2], monitoring [3,4] and improving [5-7] quality of care. Inspections were targeted based upon continuous collection of resident characteristics. Quality indicators (QIs), computed from resident-level clinical data, are aggregated to facility level and used for targeting facilities for review [8,9]. Following its implementation in the United States, the MDS has been adopted in a number of other countries. The Canadian Institute for Health Information's (CIHI) Continuing Care Reporting System is the data warehouse for MDS data from eight Canadian provinces/territories.

QIs, in raw form, are fractions derived from a numerator (number of residents with a particular outcome) and a denominator (number of residents at risk for the outcome and not otherwise excluded from the QI). QIs may include risk adjustment procedures, including restrictions or exclusions and covariate adjustment. QIs can be used by the facility to target care problems for continuous quality improvement efforts [10].

There are challenges in deriving QIs from resident assessment data. First, differences in types of residents living in facilities make direct comparisons difficult. Second, specification of the care recipient population whose outcome data reflect quality of care must be carefully considered. For example, some residents may be admitted for relatively short term rehabilitative care, short term respite care, or long term custodial care. The clinical trajectories of such different patient populations and differences in their mix across facilities lead to different expectations regarding facility QI performance. Third, many relevant NH outcome measures are rare, resulting in imprecise estimates. Finally, although the MDS is a standardized data collection instrument, facilities may measure outcomes differently or with varying sensitivity [11].

### Risk Adjustment

Adjustment for characteristics of residents permits fairer comparisons and improves identification of facilities with quality problems. Some resident characteristics increase risk of adverse outcomes independent of quality of care.

### Risk Adjustment Models

There are two main approaches to risk adjustment of nursing home QIs: stratification and indirect standardization. A third, multilevel modelling, has not yet migrated from academic to applied settings [12]. Stratification involves identification of discrete risk groups and computing QIs separately within each group (strata). Strengths of this approach include transparency and computational simplicity. Disadvantages include coarseness of adjustment. Coarse adjustment may result in residual confounding, the problem that differences across facilities may exist within broadly defined strata. Stratification also leads to QIs with small denominators, exacerbating issues of measurement precision and stability when event rates are low. Stratification was advocated by one of the first major operational systems of nursing home QIs [3,13]. Throughout this manuscript we refer to this approach as the *first generation *approach to QI scoring.

Indirect standardization develops risk adjustment using a multivariable regression approach. It compares observed to expected QI event rates across facilities. Expected rates are based on computations using the results of logistic regression models in a standard (typically the complete) sample of nursing homes. Covariates included in such a model are limited by clinical relevance, appropriateness, and general confounder selection issues [14]. Early approaches to comparing observed and expected QI event rates involved computing ratios of observed to expected proportions [15] although more recent algorithms use differences in proportions in the log odds scale [4,16,17]. Throughout this manuscript we refer to this approach as *second generation *adjusted QIs.

### A New Approach to Risk Adjustment

The overall goal of this manuscript is to presents a new method for risk adjustment (a *third generation *algorithm). Our method includes the desirable properties of existing methods. The method includes: restriction (excluding residents that are not reflective of the quality of care delivered by the nursing home, e.g., new admissions), indirect standardization (multivariable adjustment for carefully selected individual resident characteristics and exclusive of measures of process or structure) and stratification with direct standardization (i.e., it goes beyond reporting strata-specific QI scores by aggregating strata-specific scores into a single composite). Our research questions involves comparing the reliability and validity of QIs scored using the third generation method versus the second generation method.

Our original motivation for moving beyond indirect standardization was driven by two main factors. The first was the development of QIs that could be considered double-barrelled (e.g., measuring both decline and failure to improve). Our second motivation was driven by the distributional properties of many QIs. Since many outcomes are discontinuous and have a truncated distribution (e.g., level of depressive symptoms), facilities with different means at baseline will face differing rates of decline on the basis of chance. Therefore, stratification becomes an important aspect of QI development for fair comparison of facilities.

Although not a main goal of this manuscript, we also provide definitions for and evaluation of a much expanded array of nursing home QIs. The need for an expanded array of QIs is that nursing home residents represent a diverse population with heterogeneous clinical profiles. As such, the broader the conceptualisation of domains of quality, the more inclusive the operationalization of quality will be of individual patients. Moreover, fundamental validity questions for individual QIs for different purposes remain unanswered, and it is probable that different implementations of QIs within clinical domains will prove to vary in their suitability for policy and practice.

## Methods

### Design, Setting and Residents

Several data sets from the United States and Canada are used in this paper. The data source used initially to derive the 79 nursing home chronic care QIs was a 209 nursing home sample from six states (California, Illinois, Missouri, Ohio, Pennsylvania and Tennessee) covering the third quarter of 2001 and first and second quarters of 2002. This sample was created within the CMS Mega QI study [4]. The 79 QIs were based on all MDS assessments at these facilities during this period. Program process data were collected as part of the Mega QI study, details of which are reported elsewhere [4,18,19]. Participating facilities allowed trained research nurses to interview management staff, observe interactions and abstract a sample of up to 30 records. States were selected for regional representation and numbers of facilities. Facility selection was stratified based upon volume of post-hospital discharge sub-acute care provided as indicated by whether the facility was hospital based. A total of 338 free-standing facilities were approached about participating in the study and accrual was terminated after 209 agreed to participate. Data collection averaged between two and three days per facility and resulted in a validation sample size of 5,738 residents.

It is important to note that although we use data collected under previous contract work with CMS to develop our new quality indicators, the results and methods we describe here are completely original. The regression coefficients and stratification weights used in our adjustment process are not part of the previous work. Interested readers may consult the technical reports from the Mega-QI study for additional details on the sample recruitment and characteristics, and the rationale and development procedure for second-generation quality indicators. Technical details on our adjustment procedure, including computer syntax with regression and stratification weights, are available upon request.

The data source used to create the adjustment models and report on cross jurisdiction distributions of measures consists of facilities in four U.S. states and two Canadian Provinces. The work in generating these data sets and specifying this analysis framework was supported by grants and contracts from the U.S. government [20], several U.S. States [21], and the Canadian Institute for Health Information (CIHI). In each of these data sets, a full panel of MDS data were available, representing up to 3,294 U.S. facilities and 92 Canadian facilities.

### Construction of Third-Generation Quality Indicators

Our method for computing QIs extends methods derived from and currently used by CMS, that is documented in a technical report prepared by Abt Associates, Inc. [16]. It compares the proportion of observed and expected residents within a nursing home triggering the QI as differences in a log odds scale. Coefficients used in computing estimated QI scores are fixed, having been estimated from a standard data set (e.g., the full US nursing home population for a fixed period). Each QI has a unique set of covariates and restrictions. Detailed information on each of the third generation QIs is provided in Additional file 1. Computer code is available upon request.

The new *third generation *QI adjustments refine the selection of covariates and extend the stratification. First, covariates are improved through using a refined set of measures in each model relating outcomes with baseline characteristics, and introducing more powerful summary scales whenever possible (e.g., a summary measure of ADL status in place of individual ADL items). Second, we added a composite stratification variable to each QI model. Numerous candidate measures were reviewed, including summary measures of ADLs, cognition, and the Resource Utilization Groups (RUG-III) case mix algorithm. Residents are sorted into low, middle and high risk groups based on thresholds set at the 20^th ^and 80^th ^percentile of key stratification variable distribution determined from analysis of a cross national standardization sample. Details on the threshold values can be obtained by request as part of the technical specification and computer code for the QIs upon request. Indirect standardization is performed within strata. Regression coefficients for each stratum are then used to compute expected numbers of residents triggering the QI in a given facility. Finally, strata-specific expected scores are combined using weights from the standard population (see example in Additional file 2).

As with second generation QIs, inference on relative quality of care reflected by third generation adjusted QI is not placed on the absolute value of the adjusted QI, or even the value of the adjusted QI relative to the raw QI. Inference of quality is based on the QI score relative to the overall sample mean used in the standardization procedure.

For our analyses, the standard population used was represented by the large multi-state and a two-province database of resident data from nursing homes (from the U.S.) and complex continuing care hospitals/units in Canada (N~170,000). The standard population affects all facilities equally. Ideally the standard population will be broadly representative of each target facility that is the focus of quality monitoring or research question. This database was used for construction of reference population means, regression coefficients, and weights used in the adjustment procedure. Complete specification of our QIs are available in Additional file 1, computer code is available upon request.

### Assessment of Reliability

The new QIs are tested for reliability and validity. When reporting QI scores for use in surveys or internal activities, the assumption is that rates derived from assessment in the prior period reflect current status. Thus, it is important to evaluate the reliability or stability of QIs over time. Reliability was assessed with quarter-to-quarter autocorrelation coefficients.

### Assessment of Validity

#### Field Data Collection

As part of the Mega QI study, data collection in each facility included independent data describing the process of care at facility and individual resident level. Details on the validation field study are presented elsewhere [4]. We summarize aspects of the field study here. Data collection (2001-2002). Teams of trained nurse researchers visited sampled facilities, and completed Medical Record Review of the charts of residents representing the 30 most recently completed MDS assessments. Nurse researchers also completed a partial MDS 2.0 assessment on each sampled resident, and Environmental Walk Through and Resident Observation survey. In addition, an Administrative Questionnaire was delivered to administrators and/or directors of nursing. The purpose of the Medical Record Review (MRR) was to obtain information regarding the care processes and types of patient/resident assessments performed by sampled facilities on select areas. Twenty-one care areas (or quality dimensions) were reviewed during the MRR (cognitive impairment, communication, delirium, depression/mood, behavior problems, ADL improvement, ADL decline, mobility/walking, falls, anti-psychotic drugs, pain, physical restraints, feeding tubes, undernutrition/low BMI/weight loss, indwelling urinary catheter, bladder incontinence, bowel incontinence, infections, pressure sores/potential for skin breakdown, burns, abrasions, skin tears, and little or no involvement in activities). For each of these domains, nurse assessors reviewed the medical record (including nursing progress notes, physician orders and progress notes, care plans, therapy consults and notes, medication administration records, flow sheets and other interdisciplinary notes and consults) for resident care and status documentation. Assessors looked for documentation on comprehensive assessments, problems/issues, change in status (within certain time frames), referrals, treatments and nursing care plans. A MDS Supplement was used to conduct assessments on all patients in the sample, including assessment areas from the MDS in selected areas (cognitive patterns, communication/hearing patterns, mood and behavior patterns, physical functioning and structural problems, continence, disease diagnoses, health conditions, oral/nutritional status, skin conditions, activity pursuit patterns, medications, special treatment procedures, and discharge potential and overall status). The Administrative Questionnaire included questions regarding staff responsibilities, staff/resident/family involvement in care, resident status, access to specialists/consultants, clinical communication channels, staff turnover, staffing ratios, planning processes, information on the organization; and training and orientation of staff. The Environmental Walk Through/Resident Observation was used to gain an overall understanding regarding whether the facility is "resident-centered", what the "feel" of the facility, and what the nature of staff-resident interactions. A series of general environmental measures were employed to describe the responsiveness of the milieu to resident strengths, needs, and problems that include general care environment measures (e.g., nature of physical environment, communication strategies, environmental manipulation and resident interactions with staff). These measures were collected through assessment, surveillance, and observation of staff technique. The nurse researchers recorded observations three times per day.

#### Nurse Researcher Qualifications, Training and Reliability

Peer Review Organizations (PROs) in participating states were contracted to hire field data collectors, with priority for registered nurses (ultimately only 1 was not an RN) with chart review experience and experience in a long-term care setting and/or in completing the MDS Version 2.0. Nurse researchers attended a five-day training and certification program led by the Mega-QI CMS Project Officer, Steering Committee members (including two RNs), five experienced RN researchers with experience in similar data collection activities. A training manual was developed and each assessor was provided a copy. Half of the training program was devoted to training in how to conduct resident assessments using a subset of items from MDS Version 2.0. To certify competency, each trainee completed a case and met individually with the lead trainer for review. To enhance and maintain consistency in coding, project staff held weekly one-hour conference calls with the assessors during the course of data collection. Minutes of the calls. Reliability among nurse researchers was assessed by having nurses complete two paired assessments and medical reviews with their partner per facility. Agreement statistics for the MDS inter-rater reliability of nurse researchers were very good (average kappa coefficient 0.78)[4].

#### Data Quality

Data quality was ensured by using computerized assisted interviewing by trained nurse assessors, frequent teleconferences among the research nurses and project staff, and by fax-back and call-back of facility administrators. For the variables used in this analysis, data were missing data about for about 8% of data elements. Missing items were handled with mean-based single imputation. Single imputation is known to produce biased standard errors, but our inferences are based on relative differences in means and covariances among a set of QIs relative to validation scales between second and third generation QI adjustment procedures, and any bias introduced through this handling of missing data will affect both adjustment procedures equally.

#### Validation Model

As reported elsewhere [4], validation procedures for the original 21 QIs targeted by the Mega QI validation study involved expert clinical panel review of potential validation elements. Potential validation elements were categorized by quality of care construct along two dimensions: preventive and responsive. Preventive strategies represent the class of anticipatory actions that a prototypical good facility would engage in an attempt to minimize the emergence of problems (e.g., staff training and facility efforts at continuous quality improvement). Responsive strategies represent reactive actions a facility would engage in once a problem identified. By definition, facilities engaging in responsive strategies for a particular QI outcome should have higher QI scores than randomly selected facilities with lower responsive activity. Examples of responsive actions include documentation of comprehensive assessments, documentation of changes in resident status, and referrals to specialists [4].

A challenge to our secondary use of the Mega QI validation data is that our list of QIs includes more QIs than were covered in the data collection - representing refinement of the QI list by our group to improve the quality of the QIs and to extend their coverage. Many QIs may be affected by activities not captured. In addition, there has not been focused effort to decide which are appropriate preventive or responsive elements for the new and expanded set of QIs. To overcome this limitation, we identified 153 variables that were used as covariates in validation models for specific QIs summarized in the Mega QI validation report. Ninety-two (92) were responsive, 61 were preventive [4]. The included validation elements are described in an appendix to the Mega-QI final report (Mega QI Report Appendix F, accessible at http://interrai.org/user_files/images/File/CMS%20%20-%20%20Final%20Report%20Appendix%20F%2012%20NOV%202002.pdf). We used principal components analysis (PCA) to extract 10 preventive and 10 responsive summary variables from among respective arrays of individual variables (i.e., the 10 first components from the PCA for each set). Within the preventive and responsive set, individual summary variables are uncorrelated (by design). Missing data among validation elements were handled with single imputation using the method of chained linear regression equations with hot deck replacement prior to the PCA procedure [22].

The determination of validity for the QI set is based on results of three separate regression models with the adjusted facility level QI score as the dependent variable. Model 1 includes 10 orthogonal preventive components, Model 2 includes 10 orthogonal responsive elements, and Model 3 the 20 components together. Results of these models are used to classify QIs into levels of presumed validity following Morris and his colleagues [4]. The highest level of validity (Level I) is reserved for QIs where the multiple correlation coefficient from the preventive model (Model 1) is equal to or greater than 0.45 or from the combined model (Model 3) is greater than 0.55. The mid level of validity (Level II) is reserved for QIs not in Level 1 and where the multiple correlation coefficient from the preventive model is in the range [0.30, 0.45) or the from combined model is in the range (0.40, 0.55]. Remaining QIs are classed in Level III. Detailed results of these analyses are reported in Additional file 3, and summarized graphically in Additional File 4.

## Results

A limited number of representative characteristics of residents and facilities in the study are reported in Additional File 5. Overall, 19% of the sample are severely impaired in daily decision making, 29% are totally dependent in dressing, and 24% have depressive symptoms as measured by the Depression Rating Scale [23].

The third and second generation QIs had about the same magnitude of cross-sectional correlation with raw QIs (0.65 and 0.61, respectively). Autocorrelation (correlation of the QI with itself over one quarter) coefficients were about the same for third and second generation QIs (0.56 and 0.59, respectively, 95% CI on difference [-0.07, 0.00]); that is, based on a comparison of the magnitude of autocorrelation coefficients (Additional Files 6 and 7), the QIs scored with the two different algorithms cluster relatively evenly along the main diagonal. Selected QIs are labeled, including QIs with relatively high and low autocorrelation coefficients and QIs with large discrepancies between second and third generation risk adjustment procedures. Highly stable QIs are those where the underlying resident characteristic is relatively immutable (e.g., restraint use) while highly instable QIs reflecting rare or dynamic underlying resident characteristics (e.g., pressure ulcers). Some QIs in the same domain have very different stability coefficients (e.g., the presence of pain (PAI0X) is more stable than worsening pain (PAN01)).

Additional files 3 details - and additional file 4 summarizes - the results of the validation analysis. In evaluating validity, one more of the new QIs was in the Level-I (top) validity (n = 3) than were among the Mega QI set (n = 2) (detailed numerical results are available in Additional file 3). More than half of new QIs achieved a "Moderate" validation level (n = 56), including eight QIs that were considered "not valid" under the second generation adjustment methodology. Twenty-three (23) QIs were assigned to the "not validated" level.

Finally, we considered differences in the distribution of QI scores in different major care sectors. We display these results in an Additional file 8, which contains kernel density plots [24] for each QI cast as sparklines [25], and overlay functions for different political jurisdictions and clinical populations drawn form our multinational data base. Functions are shown for all facilities in our sub-sample (wide gray line), long term care (LTC) facilities in the Mega QI sample from six US states (thin gray line), Ontario, Canada LTC facilities (thin black line), Ontario rehabilitation facilities (tight dot line), Ontario complex continuing care hospitals/units (short dashed line) and Nova Scotia nursing homes (dash-dot line). This figure demonstrates the importance of choosing an appropriate reference population. Many QIs show a similar distribution across politico-clinical jurisdiction (e.g., ADL01), but others show distinct patterns (e.g., ADL06) and suggest the possibility of mixtures of facility types within jurisdiction. Therefore, it is important not only to consider how political factors (payment and reimbursement systems, and selection pressures for tertiary care centers) affect aggregate client populations and outcome statistics. The first panel of Additional file 8 shows that ADL06 (Proportion of residents who improve status on early-loss ADL functioning in dressing and personal hygiene, or remain completely independent in early-loss ADLs) is bimodal within the Ontario LTC sample. This could reflect different sub-groups of facilities within the Ontario LTC sample with fundamentally different distributions of residents being subject to the improve versus remain independent barrel of this double barrelled QI (meaning that the numerator is defined by two distinct events).

## Discussion

A third generation adjustment algorithm for nursing home QIs returns reliable and valid estimates of nursing home quality of care. It incorporates stratification (matching within strata) and covariance adjustment. The new adjustment methodology can be used to score existing QIs and new QIs developed out of related instruments such as the interRAI Long Term Care Facility (interRAI LTCF; [26,27]) and CMS's proposed MDS 3.0 revision. The new method is not appreciably more computationally intensive than that of second generation QIs, in that essentially only two additional steps - stratification and then averaging across strata - are added to the adjustment procedure.

Although on the whole the third generation QIs had slightly lower (but not significantly lower) reliability estimates relative to second generation QIs, more of the third generation QIs were rated in the top validity levels. This counter intuitive finding might suggests that lack of reliability might reflect the occurrence and ability to detect true change for the new QI set, rather than poorer measurement. A high autocorrelation does not necessarily imply that evidence of validity will be found. We noted that the autocorrelation of pain worsening was greater than that of the proportion of residents in pain, but Additional file 4 shows that we were not able to provide validation for the pain worsening QI with the third generation adjustment method. However, the proportion of residents in pain did achieve a moderate level of validity (both second and third generation adjustment models).

A number (n = 23 of 79) of QIs we constructed were not validated in our secondary analysis of the Mega QI data. However, this signals only the absence of evidence of the validity of the QIs, not that the proposed QIs are not valid. Because Mega QI validation elements were collected via chart review and care giver interview for a select and focused set of QIs, requisite preventive or responsive care practices necessary to provide validity evidence for the expansive and broad set of QIs studied here may not be represented. Conversely, finding at least moderate validity for selected QIs may identify a set where positive evidence of preventive or responsive care practices recorded in the medical record or obtained by care giver interview. Such QIs may reflect, at least in part, between-facility variability in the way care is delivered and may be considered valid for public reporting, survey, or payment uses. Some of the new QIs tap care domains outside of the detailed chart review in the original CMS validation work. Therefore, our validity evidence likely provides a lower bound. In the future, we can examine alternative reliability estimates could have been generated, for example, using split resident population correlation coefficients. We do not have prospective validation data for these QIs.

Finally, it is important to note that some limitations of QI scoring in first and second generation frameworks remain in our proposed third generation framework. Particularly, the third generation framework does not directly address the problem of low base rates (rare events). More work is needed to address this issue. One approach would be to accumulate observations over a long period of time. Typically nursing home QIs are scored quarterly, but longer periods may allow the accumulation of more events and lead to more stable estimates. The cost of such an approach is the greater time lag may not capture current quality or care practices at a given facility. A better approach may be to limit consideration of viable QIs to those that do not have a low base rate.

Beyond the issue of validity, there are limitations in the conceptualization and operationalization of nursing home quality indicators that require additional research. With few exceptions, the first, second and our new third generation quality indicators are ultimately measures of aggregate clinical state or course of a facility's residents. As such, nursing home QIs reflect only one leg of the Donabedian structure-process-outcome model for quality of medical care [28]. Whether and how nursing home QIs are risk adjusted will not remedy this. A potential consequence of this framework is nihilism with respect to the prospects of developing and using measures of nursing home quality. Perhaps no better expression of the frustration of this state of affairs can be found in Charles Phillips et al.'s review, *Where should Momma go*? [29].

A limitation of our manuscript is that we do not present information comparing the relative proportion of facilities flagged as having poor quality under second and third generation adjustment procedures. Such an effort requires establishing a benchmark or threshold against which a facility may be judged as having poor quality of care. We are unaware of thresholds having been described for the first and second generation QIs, and they have not been described for third generation QIs. In the absence of clinically based thresholds, empirically based thresholds are typically used. For example basing judgments of poor quality on the basis of the mean, median or percentile of the adjusted QIs score. Use of empirically based thresholds would not be informative for comparing second and third generation QIs.

## Conclusions

A main challenge of nursing home QIs is the issue of validity. Our view of validity includes well accepted notions of whether or not the measure adequately measures the intended construct, but extends into the use and interpretation of the statistic [30]. Unfortunately, there are no unambiguous gold standards for the assessment of nursing home quality. Rational choice of outcome domains and adjustment methods may add confidence to the derived measures, but only time and experience are capable of demonstrating the validity and usefulness of various methods for computing nursing home QIs.

Although some investigators have urged caution in over interpreting nursing home QIs [29], the validity of nursing home QIs and the potential malleability of nursing home QI scores has been demonstrated with a randomised controlled trials of a simple informational and educational interventions with the support of clinical consultation by a gerontological clinical nurse specialist [10].

Further work is needed on the development or QIs for policy purposes. Ultimately, the effective and unbiased use of QIs for quality reporting and payment systems should be preceded by significant development activities devoted to the problem of identifying appropriate reference facilities for individual facilities. One strategy could be further empirical work using facility-level matching algorithms. A second strategy might be the allowing facilities to nominate peer facilities. A procedure that merged the two would allow facilities to nominate matching facilities which were vetted as appropriate matches with empirically derived matching algorithms.

## Competing interests

The authors declare that they have no competing interests.

## Authors' contributions

RNJ carried out the analysis and drafted the manuscript. JNM conceived of the analysis and participated in the direction and interpretation of the data analysis. BEF, JPH, JWP, MK and KB participated in the design of the study. All authors contributed to the writing and read and approved the final manuscript.

## Pre-publication history

The pre-publication history for this paper can be accessed here:

http://www.biomedcentral.com/1472-6963/10/96/prepub

## Supplementary Material

Additional file 1 **Definitions of individual QIs**. This file contains the operational definition of each of the evaluated quality indicators. Click here for file

Additional file 2 **Example of Third Generation Calculation of Nursing Home Quality Indicator**. This document presents details on how a third generation nursing home quality indicator is calculated. Click here for file

Additional file 3 **Detailed numerical results from the validation analysis**. This file contains a table with the detailed results of the validation analysis. Click here for file

Additional file 4 **Figure S2 Summary of validation model analyses**. This file contains a figure summarizing the results of the validation analyses of the new third generation quality indicators relative to the Abt/CMS quality indicators. Click here for file

Additional file 5 **Table S1. Characteristics of Residents and Facilities**. This file contains a table describing the characteristics of the residents and facilities used in the analysis. Click here for file

Additional file 6 **Detailed numerical results from the reliability analysis**. This file contains a table with the results of the reliability analysis (quarter to quarter autocorrelation) for the second and third generation quality indicators. Click here for file

Additional file 7 **Figure S1 Scattergraph of autocorrelation coefficients for third and second generation quality indicators**. This file contains a figure illustrating the correlation of autocorrelation coefficients for third and second generation quality indicators. Click here for file

Additional file 8 **Density plots of each quality indicator**. This document contains a density plot for each quality indicator for each major care sector. Click here for file
