# Supplementary material for: Adjustment of nursing home quality indicators
Source: BMC Health Serv Res. 2010 Apr 15;10:96. doi: 10.1186/1472-6963-10-96 (PMC2881673; doi:10.1186/1472-6963-10-96)
Supplement: Additional file 1 — Definitions of individual QIs. This file contains the operational definition of each of the evaluated quality indicators. [file 1472-6963-10-96-S1.DOC]

**Additional File 1**

**Table 1. Resident and Facility Characteristics**

**Facility Facilities**

**Characteristic (N) %**

Total 209 100

State

California 31 15

Illinois 41 20

Missouri 27 13

Ohio 33 16

Pennsylvania 45 22

Tennessee 32 15

Location

Free-standing 150 72

Hospital-based 59 28

**Sampled Resident Residents**

**Characteristics (N) %**

Total 5,738 100

Severely Impaired

daily decision making 1,113 20

Total dependence in

Dressing 1,679 29

Probable depression

(DRS≥2) 1,361 24

Note: DRS, Depression Rating Scale ^26^.
