# Supplementary material for: Adjustment of nursing home quality indicators
Source: BMC Health Serv Res. 2010 Apr 15;10:96. doi: 10.1186/1472-6963-10-96 (PMC2881673; doi:10.1186/1472-6963-10-96)
Supplement: Additional file 2 — Example of Third Generation Calculation of Nursing Home Quality Indicator. This document presents details on how a third generation nursing home quality indicator is calculated. [file 1472-6963-10-96-S2.PDF]

Additional File 2
QI Technical Definitions

Institute for Aging Research
Hebrew SeniorLife
Boston, MA

August 28, 2009

Contents

1 ADL01 – Percent of Residents who had an unexpected loss of function in some basic daily activities 7

2 ADL02 – Percent of Residents with worsening function in some basic daily activities 8

3 ADL03 – Percent of residents who have improved in their ability to function 9

4 ADL05 – Percent of Residents who improve status on mid-loss ADL functioning (transfer, locomotion) or remain completely independent in mid-loss ADLs 10

5 ADL06 – Percent of Residents who improve status on early-loss ADL functioning (dressing and personal hygiene) or remain completely independent in early-loss ADLs (ELADL). 11

6 ADL07 – Percent of Residents with ADL changes that are improvement 12

7 ADL1A – Percent of Residents who had an improvement of function in some basic daily activities 13

8 ADL5A – Percent of Residents who declined status on mid-loss ADL functioning (transfer, locomotion) 14

9 ADL6A – Percent of Residents who declined status on early-loss ADL functioning (dressing and personal hygiene) 15

10 ADLD1 – Percent of Residents who have declined in some basic daily activities 16

11 ADLD5 – Percent of Residents who have declined mid-loss ADLs 17

12 ADLD6 – Percent of Residents who have declined early-loss ADLs 18

13 ADLD7 – Percent of Residents who have declined in ADLs (ADL Long Form) 19

14 ADLI1 – Percent of Residents who have improved in some basic daily activities 20

15 ADLI5 – Percent of Residents who have improved mid-loss ADLs 21

16 ADLI6 – Percent of Residents who have improved early-loss ADLs 22

17 ADLI7 Proportion of ADLs changes that are improvement 23

18 BEH01 – Percent of Residents with inappropriate behavior 24

19 BEH04 – Percent of Residents whose behavior has worsened 25

20 BEH4A – Percent of Residents whose behavior has improved 26

21 BEHD4 – Percent of Residents who have declining behavioral symptoms 27

22 BEHI4 – Percent of Residents who have improved behavioral symptoms 28

23 BMI0X – Percent of Residents with a low BMI 29

24 BUR0X – Percent of Residents with burns, skin tears or cuts 30

25 CAT01 – Percent of Residents with a new indwelling catheter 31

26 CAT02 – Percent of Residents with indwelling catheters 32

27 CNT01 – Percent of Residents who are bladder or bowel incontinent 33

28 CNT02 – Percent of Residents with worsening bowel continence 34

29 CNT03 – Percent of Residents with worsening bladder continence 35

30 CNT04 – Percent of Residents with a urinary tract infection 36

31 CNT2A – Percent of Residents with improving bowel continence 37

32 CNT3A – Percent of Residents with improving bladder continence 38

33 CNTD2 – Percent of Residents who have declined bowel continence 39

34 CNTD3 – Percent of Residents who have declined bladder continence 40

35 CNTI2 – Percent of Residents who have improved bowel continence 41

36 CNTI3 – Percent of Residents who have improved bladder continence 42

37 CNTX2 – Percent of Residents who failed to improve their bladder incontinence 43

38 COG01 – Percent of Residents whose cognitive ability has worsened 44

39 COG1A – Percent of Residents whose cognitive ability has improved 45

40 COGD1 – Percent of Residents whose ability to communicate has declined 46

41 COGI1 – Percent of Residents who have improved cognitive performance 47

42 COM01 – Percent of Residents whose ability to communicate has worsened 48

43 COM1A – Percent of Residents whose ability to communicate has improved 49

44 COMD1 – Percent of Residents whose ability to communicate has worsened 50

45 COMI1 – Percent of Residents who have improved communication 51

46 DEL0X – Percent of Residents with symptoms of delirium 52

47 DRG01 – Percent of Residents on antipsychotics without a diagnosis of psychosis 53

48 FAL01 – Percent of Residents who have fallen 54

49 FAL02 – Percent of Residents who have fallen in the last 30-days 55

50 INF0X – Percent of Residents with infections 56

51 MOB01 – Percent of Residents who have declined in their ability to locomote 57

52 MOB1A – Percent of Residents who have improved in their ability to locomote 58

53 MOBD1 – Percent of Residents who have declined in their ability to locomote 59

54 MOBI1 – Percent of Residents who have improved in their ability to locomote 60

55 MOD03 – Percent of Residents who have become more depressed or anxious 61

56 MOD04 – Percent of Residents who improve their mood or remain free from symptoms of depression (based on MDS Depression Rating Scale) 62

57 MOD4A – Percent of Residents who decline in mood from symptoms of depression (based on MDS Depression Rating Scale) 63

58 MODD4 – Percent of Residents who decline in mood from symptoms of depression (based on MDS Depression Rating Scale) 64

59 MODI4 – Percent of Residents who improved in mood from symptoms of depression (based on MDS Depression Rating Scale) 65

60 NUT01 – Percent of Residents with a feeding tube 66

61 PAI0X – Percent of Residents with pain 67

62 PAN01 – Percent of Residents with worsening pain 68

63 PAN1A – Percent of Residents with improving pain 69

64 PAND1 – Percent of Residents who have declined in pain 70

65 PANI1 – Percent of Residents who have improved pain 71

66 PRU01 – Percent of Residents with pressure sores 72

67 PRU04 – Percent of Residents with worsening pressure sores 73

68 PRU05 – Percent of Residents who have a Pressure Ulcer Stage 2 to 4 74

69 PRU09 – Percent of Residents who have a newly ocurring pressure ulcer stage 2 to 4 75

70 PRU4A – Percent of Residents with improving pressure sores 76

71 PRUD4 – Percent of Residents who have declined pressure ulcer 77

72 PRUI4 – Percent of Residents who have improved pressure ulcer 78

73 RES01 – Percent of Residents in physical restraints 79

74 RSP02 – Percent of Residents who do not have shortness of breath 80

75 RSPX2 – Percent of Residents who have developed a respiratory infection or have not gotten better 81

76 SOC02 – Percent of Residents engaging in little or no activity 82

77 WAL0X – Percent of Residents who walk as well or better than the previous assessment 83

78 WALX1 – Percent of Residents who walk as well or better on target assessment as on the prior assessment of their stay. 84

79 WGT01 – Percent of Residents who have unexplained weight loss 85

80 Scales 86

1 ADL01 – Percent of Residents who had an unexpected loss of function in some basic daily activities

NUMERATOR:
Residents with worsening (increasing item score) in Late-Loss ADL self-performance at target relative to prior assessment. Residents meet the definition of Late-Loss ADL worsening when at least two of the following are true:
1. G1a(A)[t]-G1a(A)[t-1] > 0, or 2. G1b(A)[t]-G1b(A)[t-1] > 0, or 3. G1h(A)[t]-G1h(A)[t-1] > 0, or 4. G1i(A)[t]-G1i(A)[t-1] > 0,
OR at least one of the following is true: 1. G1a(A)[t]-G1a(A)[t-1] > 1, or 2. G1b(A)[t]-G1b(A)[t-1] > 1, or 3. G1h(A)[t]-G1h(A)[t-1] > 1, or 4. G1i(A)[t]-G1i(A)[t-1] > 1.
Note: Late-Loss ADL items values of 8 are recoded to 4 for evaluation of change.

DENOMINATOR:
All residents with a valid target and a valid prior assessment.

COVARIATE(S):
Age less than 65

EXCLUSION(S):
All Medicare Assessments coded in AA8B
Two Consecutive Chronic Admission Assessments
None of the four Late-Loss ADLs (G1a(A), G1b(A), G1h(A), and G1i(A)) can show decline because each of the four have a value of 4 (total dependence) or a value 8 (activity did not occur) on the prior assessment [t-1].
There is missing data on any one of the four Late-Loss ADLs (G1a(A), G1b(A), G1h(A), or G1i(A)) on the target assessment [t] or prior assessment [t-1].
The resident is comatose (B1 = 1) or comatose status is unknown (B1 = missing) on the target assessment.
The resident has end-stage disease (J5c = checked) or end-stage disease status is unknown (J5c = missing) on the target assessment.
The resident is receiving hospice care (P1ao = checked) or hospice status is unknown (P1ao = missing) on the target assessment.

STRATIFICATION:
ADL-Long

2 ADL02 – Percent of Residents with worsening function in some basic daily activities

NUMERATOR:
Residents with ADL Long Form score (ADLLF) showing worsening (increasing ADLLF score) following improvement (decreasing ADLLF score): assessments) $\text{ADLLF[t]} > \text{ADLLF[t-1]} < \text{ADLLF[t-2]}$

DENOMINATOR:
All residents with a valid target assessment, a valid prior assessment, and a valid prior-1 assessment.

COVARIATE(S):
Age less than 65

EXCLUSION(S):
All Medicare Assessments coded in AA8B
The ADLLF score is missing on either the target [t] or the prior [t-1] assessment.
The ADLLF score is missing on the prior-1 assessment [t-2] and the ADLLF score is below the ceiling on the prior assessment (ADLLF[t-1] $<$ 28.
The ADLLF score is at the scale floor on the prior-1 assessment (ADLLF[t-2] = 0).
The ADLLF score is at the scale ceiling on the prior assessment (ADLLF[t-1] = 28).

STRATIFICATION:
CMI

3 ADL03 – Percent of residents who have improved in their ability to function

NUMERATOR:
Residents with ADL Long Form scale (ADLLF) lower at target assessment relative to prior assessment, implying improvement (ADLLF[t] < ADLLF[t- 1]).

DENOMINATOR:
All residents with a valid target assessment and a valid prior assessment AND with any
one of the following Inclusion Indicators of ADL functional rehabilitation capacity in items G8a through G8d on the most recent full assessment:
1. Resident believes there is capability of increased independence (G8a = checked).
2. Staff believes there is capability of increased independence (G8b = checked).
3. Resident able to perform tasks/activity but is very slow (G8c = checked).
4. Difference in ADL self -performance or support comparing mornings to evenings (G8d = checked).

COVARIATE(S):
Age less than 65
Cognitive Performance Scale

EXCLUSION(S):
All Medicare Assessments coded in AA8B
Two Consecutive Chronic Admission Assessments
Residents satisfying any of the following conditions:
The ADLLF score is missing on the target assessment [t].
The ADLLF score is missing on the prior assessment [t-1] and the ADLLF score is below the ceiling on the target assessment (ADLLF[t] < 28).
The ADLLF score is at the scale floor on the prior assessment (ADLLF[t-1] = 0).
The resident is comatose (B1 = 1) or comatose status is unknown (B1 = missing) on the target assessment.
The resident has end-stage disease (J5c = checked) or status is unknown (J5c = missing) on the target assessment.
The resident is receiving hospice care (P1ao = checked) or hospice status is unknown (P1ao = missing) on the target assessment.

STRATIFICATION:
CMI

4 ADL05 – Percent of Residents who improve status on mid-loss ADL functioning (transfer, locomotion) or remain completely independent in mid-loss ADLs

NUMERATOR:
Residents with a MLADL change score that is negative (MLADL[t]-MLADL[t-1]<0) OR
2. Residents with a MLADL score of 0 at Prior and Target assessments (MLADL[t]=0 AND MLADL[t-1]=0).
MLADL is defined as the sum of G1b(A), G1e(A) and G1d(A), with 8's recoded to 4's)

DENOMINATOR:
All residents with a valid target assessment and a valid prior assessment

COVARIATE(S):
Age less than 65
Cognitive Performance Scale

EXCLUSION(S):
All Medicare Assessments coded in AA8B
Two Consecutive Chronic Admission Assessments
Residents satisfying any of the following conditions:
Comatose (B1 = 1) or comatose status unknown (B1 = missing) on Target assessment
End stage disease (J5c=checked) or end stage disease status unknown (J5c = missing) on Target assessment
Hospice (P1ao = checked) or hospice status unknown (P1ao = missing) on Target assessment
Residents with non-valid Mid-Loss ADL at the Target assessment (MLADL[t]=missing)
Residents with non-valid Mid-Loss ADL at the Prior assessment (MLADL[t-1] = missing) AND MLADL is greater than 0 at the Target assessment (MLADL[t]>0)

STRATIFICATION:
ADL-Long

5 ADL06 – Percent of Residents who improve status on early-loss ADL functioning (dressing and personal hygiene) or remain completely independent in early-loss ADLs (ELADL).

NUMERATOR:
Residents with a ELADL change score that is negative (ELADL[t]-ELADL[t-1]<0) OR
2. Residents with a ELADL score of 0 at Prior and Target assessments (ELADL[t]=0 AND ELADL[t-1]=0).
ELADL is defined as the sum of G1g(A) and G1j(A), with 8's recoded to 4's

DENOMINATOR:
All residents with a valid target assessment and a valid prior assessment

COVARIATE(S):
RUG Late Loss ADL Scale
Age less than 65

EXCLUSION(S):
All Medicare Assessments coded in AA8B
Two Consecutive Chronic Admission Assessments
Residents satisfying any of the following conditions:
Comatose (B1 = 1) or comatose status unknown (B1 = missing) on Target assessment
End stage disease (J5c=checked) or end stage disease status unknown (J5c = missing) on Target assessment
Hospice (P1ao = checked) or hospice status unknown (P1ao = missing) on Target assessment
Residents with non-valid Early Loss ADL at the Target assessment (ELADL[t] = missing)
Residents with non-valid Early-Loss ADL at the Prior assessment (ELADL[t-1] = missing) AND ELADL is greater than 0 at the Target assessment (ELADL[t]>0)

STRATIFICATION:
CPS

6 ADL07 – Percent of Residents with ADL changes that are improvement

NUMERATOR:
Residents with a valid ADL-Long scale at target assessment less than at prior assessment

DENOMINATOR:
All residents with a valid target assessment and a valid prior assessment.

COVARIATE(S):
Age less than 65

EXCLUSION(S):
All Medicare Assessments coded in AA8B
Two Consecutive Chronic Admission Assessments
The resident is comatose (B1=1,yes)
The resident has end-stage disease (J5c = checked)
Hospice (P1ao = checked)
Any Item in ADL-Long scale is missing on either the target [t] or the prior [t-1] assessment.

STRATIFICATION:
ADL-Long

7 ADL1A – Percent of Residents who had an improvement of function in some basic daily activities

NUMERATOR:
Residents with improvement (decreasing item score) in Late-Loss ADL self-performance at target relative to prior assessment. Residents meet the definition of Late-Loss ADL worsening when at least two of the following are true:
OR at least one of the following is true: 1. G1a(A)[t]-G1a(A)[t-1] <-1, or 2. G1b(A)[t]-G1b(A)[t-1] <-1, or 3. G1h(A)[t]-G1h(A)[t-1] <-1, or 4. G1i(A)[t]-G1i(A)[t-1] <-1.
OR at least one of the following is true: 1. G1a(A)[t]-G1a(A)[t-1] < 0, or 2. G1b(A)[t]-G1b(A)[t-1] < 0, or 3. G1h(A)[t]-G1h(A)[t-1] < 0, or 4. G1i(A)[t]-G1i(A)[t-1] < 0.
Note: Late-Loss ADL items values of 8 are recoded to 4 for evaluation of change.

DENOMINATOR:
All residents with a valid target and a valid prior assessment.

COVARIATE(S):
PSI: Subset 1 Diagnoses
Cognitive Performance Scale
RUG Behavior
RUG Cognitive Impairment
Age less than 65

EXCLUSION(S):
All Medicare Assessments coded in AA8B
Two Consecutive Chronic Admission Assessments
The resident is comatose (B1 = 1) or comatose status is unknown (B1= missing) on the target assessment.
End stage disease (J5c=checked) or end stage disease status unknown (J5c = missing) on Target assessment
Hospice (P1ao = checked) or hospice status unknown (P1ao = missing) on Target assessment
None of the four Late-Loss ADLs (G1a(A), G1b(A), G1h(A), and G1i(A)) can show improvement because each of the four have a value of 0 (independence) on the prior assessment [t-1]
There is missing data on any one of the four Late-Loss ADLs (G1a(A), G1b(A), G1h(A), or G1i(A)) on the target assessment [t] or prior assessment [t-1].

STRATIFICATION:
CMI

8 ADL5A – Percent of Residents who declined status on mid-loss ADL functioning (transfer, locomotion)

NUMERATOR:
Residents with a MLADL change score that is positive (MLADL[t]-MLADL[t-1]>0)
MLADL is defined as the sum of G1b(A), G1e(A) and G1d(A), with 8's recoded to 4's)

DENOMINATOR:
All residents with a valid target assessment and a valid prior assessment

COVARIATE(S):
Not totally dependent in transferring
Locomotion problem
PSI: Subset 2 Non-Diagnoses
Age less than 65

EXCLUSION(S):
All Medicare Assessments coded in AA8B
Two Consecutive Chronic Admission Assessments
The resident is comatose (B1=1,yes)
The resident has end-stage disease (J5c = checked)
4. The resident has end-stage disease (J5c = checked)
4. The resident has end-stage disease (J5c = checked)
Hospice (P1ao = checked)
MLADL is missing at the target assessment
MADL is missing at the prior assessment and MADl is gt 0 at the target assessment
MLADL is 12 [unable to decline further] at the prior assessment.

STRATIFICATION:
CMI

9 ADL6A – Percent of Residents who declined status on early-loss ADL functioning (dressing and personal hygiene)

NUMERATOR:
Residents with a ELADL change score that is positive (ELADL[t]-ELADL[t-1]>0)
ELADL is defined as the sum of G1g(A) and G1j(A), with 8's recoded to 4's

DENOMINATOR:
All residents with a valid target assessment and a valid prior assessment.

COVARIATE(S):
Not totally dependent in transferring
Locomotion problem
PSI: Subset 2 Non-Diagnoses
Cognitive Performance Scale
Age less than 65

EXCLUSION(S):
All Medicare Assessments coded in AA8B
Two Consecutive Chronic Admission Assessments
The resident is comatose (B1=1,yes)
The resident has end-stage disease (J5c = checked)
Hospice (P1ao = checked)
ELADL is missing at the target assessment
EADL is missing at the prior assessment and EADL is gt 0 at the target assessment
ELADL is 8 [unable to decline further] at the prior assessment

STRATIFICATION:
CMI

10 ADLD1 – Percent of Residents who have declined in some basic daily activities

NUMERATOR:
Residents with worsening (increasing item score) in Late-Loss ADL self-performance at target relative to prior assessment. Residents meet the definition of Late-Loss ADL worsening when at least two of the following are true:
1. G1a(A)[t]-G1a(A)[t-1] > 0, or 2. G1b(A)[t]-G1b(A)[t-1] > 0, or 3. G1h(A)[t]-G1h(A)[t-1] > 0, or 4. G1i(A)[t]-G1i(A)[t-1] > 0,
OR at least one of the following is true: 1. G1a(A)[t]-G1a(A)[t-1] > 1, or 2. G1b(A)[t]-G1b(A)[t-1] > 1, or 3. G1h(A)[t]-G1h(A)[t-1] > 1, or 4. G1i(A)[t]-G1i(A)[t-1] > 1.
Note: Late-Loss ADL items values of 8 are recoded to 4 for evaluation of change.

DENOMINATOR:
All residents with a valid target assessment and a valid prior assessment.

COVARIATE(S):
Not totally dependent in transferring
Locomotion problem
PSI: Subset 2 Non-Diagnoses
Age less than 65

EXCLUSION(S):
All Medicare Assessments coded in AA8B
Two Consecutive Chronic Admission Assessments
The resident is comatose (B1=1,yes)
The resident has end-stage disease (J5c = checked)
Hospice (P1ao = checked)

STRATIFICATION:
ADL-Long

11 ADLD5 – Percent of Residents who have declined mid-loss ADLs

NUMERATOR:
Residents with a MLADL change score that is negative (MLADL[t]-MLADL[t-1]<0) OR
2. Residents with a MLADL score of 0 at Prior and Target assessments (MLADL[t]=0 AND MLADL[t-1]=0).
MLADL is defined as the sum of G1b(A), G1e(A) and G1d(A), with 8's recoded to 4's)

DENOMINATOR:
All residents with a valid target assessment and a valid prior assessment.

COVARIATE(S):
Not totally dependent in transferring
Locomotion problem
PSI: Subset 2 Non-Diagnoses
Age less than 65

EXCLUSION(S):
All Medicare Assessments coded in AA8B
Two Consecutive Chronic Admission Assessments
The resident is comatose (B1=1,yes)
The resident has end-stage disease (J5c = checked)
Hospice (P1ao = checked)

STRATIFICATION:
CMI

12 ADLD6 – Percent of Residents who have declined early-loss ADLs

NUMERATOR:
Residents with a ELADL change score that is positive (ELADL[t]-ELADL[t-1]>0)
ELADL is defined as the sum of G1g(A) and G1j(A), with 8's recoded to 4's

DENOMINATOR:
All residents with a valid target assessment and a valid prior assessment.

COVARIATE(S):
Not totally dependent in transferring
Locomotion problem
PSI: Subset 2 Non-Diagnoses
Cognitive Performance Scale
Age less than 65

EXCLUSION(S):
All Medicare Assessments coded in AA8B
Two Consecutive Chronic Admission Assessments
The resident is comatose (B1=1,yes)
The resident has end-stage disease (J5c = checked)
Hospice (P1ao = checked)

STRATIFICATION:
CMI

13 ADLD7 – Percent of Residents who have declined in ADLs (ADL Long Form)

NUMERATOR:
Residents with a valid ADL-Long scale at target assessment greater than at prior assessment

DENOMINATOR:
All residents with a valid target assessment and a valid prior assessment.

COVARIATE(S):
Not totally dependent in transferring
Locomotion problem
PSI: Subset 2 Non-Diagnoses
Age less than 65

EXCLUSION(S):
All Medicare Assessments coded in AA8B
Two Consecutive Chronic Admission Assessments
The resident is comatose (B1=1,yes)
The resident has end-stage disease (J5c = checked)
Hospice (P1ao = checked)
Any Item in ADL-Long scale is missing on either the target [t] or the prior [t-1] assessment.

STRATIFICATION:
CMI

14 ADLI1 – Percent of Residents who have improved in some basic daily activities

NUMERATOR:
Residents with improvement (decreasing item score) in Late-Loss ADL self-performance at target relative to prior assessment. Residents meet the definition of Late-Loss ADL worsening when at least two of the following are true:
OR at least one of the following is true: 1. G1a(A)[t]-G1a(A)[t-1] < 1, or 2. G1b(A)[t]-G1b(A)[t-1] < 1, or 3. G1h(A)[t]-G1h(A)[t-1] < 1, or 4. G1i(A)[t]-G1i(A)[t-1] < 1.
OR at least one of the following is true: 1. G1a(A)[t]-G1a(A)[t-1] < 1, or 2. G1b(A)[t]-G1b(A)[t-1] < 1, or 3. G1h(A)[t]-G1h(A)[t-1] < 1, or 4. G1i(A)[t]-G1i(A)[t-1] < 1.
Note: Late-Loss ADL items values of 8 are recoded to 4 for evaluation of change.

DENOMINATOR:
All residents with a valid target assessment and a valid prior assessment.

COVARIATE(S):
RUG Behavior
RUG Cognitive
PSI: Subset 1 Diagnoses
Cognitive Perfomance Scale
Age less than 65

EXCLUSION(S):
All Medicare Assessments coded in AA8B
Two Consecutive Chronic Admission Assessments
The resident is comatose (B1=1,yes)
The resident has end-stage disease (J5c = checked)
Hospice (P1ao = checked)

STRATIFICATION:
CMI

15 ADLI5 – Percent of Residents who have improved mid-loss ADLs

NUMERATOR:
Residents with a MLADL change score that is negative (MLADL[t]-MLADL[t-1]<0) OR
Residents with a MLADL score of 0 at Prior and Target assessments (MLADL[t]=0 AND MLADL[t-1]=0).
MLADL is defined as the sum of G1b(A), G1e(A) and G1d(A), with 8's recoded to 4's)

DENOMINATOR:
All residents with a valid target assessment and a valid prior assessment.

COVARIATE(S):
PSI: Subset 2 Non-Diagnoses
Cognitive Performance Scale
Requires much assistance for eating
Not totally dependent in transferring
Age less than 65

EXCLUSION(S):
All Medicare Assessments coded in AA8B
Two Consecutive Chronic Admission Assessments
The resident is comatose (B1=1,yes)
The resident has end-stage disease (J5c = checked)
Hospice (P1ao = checked)

STRATIFICATION:
CPS

16 ADLI6 – Percent of Residents who have improved early-loss ADLs

NUMERATOR:
Residents with a ELADL change score that is negative (ELADL[t]-ELADL[t-1]<0) OR
2. Residents with a ELADL score of 0 at Prior and Target assessments (ELADL[t]=0 AND ELADL[t-1]=0).
ELADL is defined as the sum of G1g(A) and G1j(A), with 8's recoded to 4's

DENOMINATOR:
All residents with a valid target assessment and a valid prior assessment.

COVARIATE(S):
Full PSI
PSI: Subset 2 Non-Diagnosis
Cognitive Perfomance Scale
More Dependence in Toileting
Requires much assistance for eating
Locomotion problem
Not totally dependent in transferring
Age less than 65

EXCLUSION(S):
All Medicare Assessments coded in AA8B
Two Consecutive Chronic Admission Assessments
The resident is comatose (B1=1,yes)
The resident has end-stage disease (J5c = checked)
Hospice (P1ao = checked)

STRATIFICATION:
CPS

17 ADLI7 Proportion of ADLs changes that are improvement

NUMERATOR:
Residents with a valid ADL-Long scale at target assessment less than at prior assessment

DENOMINATOR:
All residents with a valid target assessment and a valid prior assessment.

COVARIATE(S):
PSI: Subset 2 Non-Diagnoses
Cognitive Perfomance Scale
Requires much assistance for eating
Age less than 65

EXCLUSION(S):
All Medicare Assessments coded in AA8B
Two Consecutive Chronic Admission Assessments
The resident is comatose (B1=1,yes)
The resident has end-stage disease (J5c = checked)
Hospice (P1ao = checked)
Any Item in ADL-Long scale is missing on either the target [t] or the prior [t-1] assessment.

STRATIFICATION:
CPS

18 BEH01 – Percent of Residents with inappropriate behavior

NUMERATOR:
Residents with behavioral symptoms affecting others on target assessment, including any verbally abusive behavior (E4b(A)>0), physically abusive behavior (E4c(A)>0) or socially inappropriate behavior (E4d(A)>0).

DENOMINATOR:
All residents with a valid target assessment.

COVARIATE(S):
RUG Cognitive Impairment
Cognitive Performance Scale
Long term memory problem
Age less than 65

EXCLUSION(S):
All Medicare Assessments coded in AA8B
Admission Assessment at the Target Assessment
Residents satisfying any of the following conditions are excluded:
The resident is comatose (B1 = 1) or comatose status is unknown (B1= missing) on the target assessment.
The value of E4b(A), E4c(A), or E4d(A) is missing on the target assessment.

STRATIFICATION:
DRS

19 BEH04 – Percent of Residents whose behavior has worsened

NUMERATOR:
Residents with more behavioral symptoms present at target assessment ([t]) relative to prior assessment ([t-1]).
Included symptoms are Wandering (E4a(A)>0), Verbally abusive behavior (E4b(A)>0), Physically abusive behavior (E4c(A)>0), and Socially inappropriate behavior (E4d(A)>0).
Specifically: Count(E4a(A) > 0, E4b(A) > 0, E4c(A) > 0, E4d(A) > 0) at time [t] > Count(E4a(A) > 0, E4b(A) > 0, E4c(A) > 0, E4d(A) > 0) at time [t-1]

DENOMINATOR:
All residents with a valid target assessment and a valid prior assessment.

COVARIATE(S):
Age less than 65

EXCLUSION(S):
All Medicare Assessments coded in AA8B
Two Consecutive Chronic Admission Assessments
All four behavior symptoms are present on the prior assessment: E4a(A) > 0 AND E4b(A) > 0 AND E4c(A) > 0 AND E4d(A) > 0.
Any of the four behavior items (E4a(A), E4b(A), E4c(A), E4d(A)) are missing on the target assessment [t] or prior assessment [t-1].
The resident is comatose (B1 = 1) or comatose status is unknown (B1= missing) on the target assessment

STRATIFICATION:
CPS

20 BEH4A – Percent of Residents whose behavior has improved

NUMERATOR:
Residents with less behavioral symptoms present at target assessment ([t]) relative to prior assessment ([t-1]).
Included symptoms are Wandering (E4a(A)>0), Verbally abusive behavior (E4b(A)>0), Physically abusive behavior (E4c(A)>0), and Socially inappropriate behavior (E4d(A)>0).
Specifically: Count(E4a(A) > 0, E4b(A) > 0, E4c(A) > 0, E4d(A) > 0) at time [t] > Count(E4a(A) > 0, E4b(A) > 0, E4c(A) > 0, E4d(A) > 0) at time [t-1]

DENOMINATOR:
All residents with a valid target assessment and a valid prior assessment.

COVARIATE(S):
Age less than 65
Motor Agitation
Moderate/impaired decision making problem

EXCLUSION(S):
All Medicare Assessments coded in AA8B
Two Consecutive Chronic Admission Assessments
All four behavior symptoms are present on the prior assessment: E4a(A) > 0 AND E4b(A) > 0 AND E4c(A) > 0 AND E4d(A) > 0.
Any of the four behavior items (E4a(A), E4b(A), E4c(A), E4d(A)) are missing on the target assessment [t] or prior assessment [t-1].
The resident is comatose (B1 = 1) or comatose status is unknown (B1= missing) on the target assessment

STRATIFICATION:
CPS

21 BEHD4 – Percent of Residents who have declining behavioral symptoms

NUMERATOR:
Residents with more behavioral symptoms present at target assessment ([t]) relative to prior assessment ([t-1]).
Included symptoms are Wandering (E4a(A)>0), Verbally abusive behavior (E4b(A)>0), Physically abusive behavior (E4c(A)>0), and Socially inappropriate behavior (E4d(A)>0).
Specifically: Count(E4a(A) > 0, E4b(A) > 0, E4c(A) > 0, E4d(A) > 0) at time [t] > Count(E4a(A) > 0, E4b(A) > 0, E4c(A) > 0, E4d(A) > 0) at time [t-1]

DENOMINATOR:
All residents with a valid target assessment and a valid prior assessment.

COVARIATE(S):
Cognitive Performance Scale
Motor Agitation
Age less than 65

EXCLUSION(S):
All Medicare Assessments coded in AA8B
Two Consecutive Chronic Admission Assessments
Any of the four behavior items (E4a(A), E4b(A), E4c(A), E4d(A)) are missing on the target assessment [t]
The resident is comatose (B1 = 1)

STRATIFICATION:
CPS

22 BEHI4 – Percent of Residents who have improved behavioral symptoms

NUMERATOR:
Residents with a valid behavioral symptom items [E4aa,E4ba,E4ca,E4da] at the target assessment and at prior assessment
Included symptoms are Wandering (E4a(A)>0), Verbally abusive behavior (E4b(A)>0), Physically abusive behavior (E4c(A)>0), and Socially inappropriate behavior (E4d(A)>0).
Specifically: Count(E4a(A) $>$ 0, E4b(A) $>$ 0, E4c(A) $>$ 0, E4d(A) $>$ 0) at time [t] $>$ Count(E4a(A) $>$ 0, E4b(A) $>$ 0, E4c(A) $>$ 0, E4d(A) $>$ 0) at time [t-1]

DENOMINATOR:
All residents with a valid target assessment and a valid prior assessment.

COVARIATE(S):
Moderate/impaired decision making problem
Motor Agitation
Age less than 65

EXCLUSION(S):
All Medicare Assessments coded in AA8B
Two Consecutive Chronic Admission Assessments
Any of the four behavior items (E4a(A), E4b(A), E4c(A), E4d(A)) are missing at target assessment
The resident is comatose (B1 = 1)

STRATIFICATION:
CPS

23 BMI0X – Percent of Residents with a low BMI

NUMERATOR:
Total number of residents with Body Mass Index (BMI) less than or equal to 19kg/m2 on the target
assessment or most recent full assessment (only if the most recent full assessment is a non-admission
assessment with AA8a = 02, 03, or 04).
$BMI = weight(kg)/height(m)^2 = ((K2b * 0.45)/(((K2a) * .0254)^2))$

DENOMINATOR:
All residents with a valid target assessment.

COVARIATE(S):
Age less than 65
RUG Nursing CMI

EXCLUSION(S):
All Medicare Assessments coded in AA8B
Admission Assessment at the Target Assessment
Residents satisfying any of the following conditions:
The selected value for either K2a or K2b from the target assessment or most recent full assessment is
missing.
The computed BMI value is outside of a plausible range. The BMI value is < 12 OR > 40.
The resident is comatose (B1 = 1) or comatose status is unknown (B1 = missing) on the target assessment
Resident has end-stage disease (J5c = checked) or the status of end-stage disease is unknown (J5c =
missing) on the target assessment.
The resident is receiving hospice care (P1ao = checked) or hospice status is unknown (P1ao =
missing) on the target assessment.

STRATIFICATION:
ADL-Long

24 BUR0X – Percent of Residents with burns, skin tears or cuts

NUMERATOR:
Total number of residents who have burns, skin tears, or cuts (M4b = checked OR M4f = checked) on the target or most recent full assessment (only if the most recent full assessment is a non-admission assessment with AA8a = 02, 03, or 04).

DENOMINATOR:
All residents with a valid target assessment.

COVARIATE(S):
Age less than 65

EXCLUSION(S):
All Medicare Assessments coded in AA8B
Admission Assessment at the Target Assessment
Any M4b or M4f has a missing value on the target assessment.

STRATIFICATION:
ADL-Long

25 CAT01 – Percent of Residents with a new indwelling catheter

NUMERATOR:
Residents with an indwelling catheter (H3d[t]=checked) on the target assessment that did not have an indwelling catheter at prior assessment (H3d[t-1]= not checked).

DENOMINATOR:
All residents with a valid target assessment and a valid prior assessment.

COVARIATE(S):
Age less than 65

EXCLUSION(S):
All Medicare Assessments coded in AA8B
Two Consecutive Chronic Admission Assessments
Residents satisfying the following condition:
H3d is missing on either the target assessment or the prior assessment.
The resident has end-stage disease (J5c = checked) or status is unknown (J5c = missing) on the target assessment.
The resident is receiving hospice care (P1ao = checked) or hospice status is unknown (P1ao = missing) on the target assessment.

STRATIFICATION:
CMI

26 CAT02 – Percent of Residents with indwelling catheters

NUMERATOR:
Resdients with an Indwelling catheter on target assessment (H3d[t]=checked).

DENOMINATOR:
All residents with a valid target assessment.

COVARIATE(S):
Pressure Sore (stage 3 or 4)
ALS/MS Diagnosis
Age less than 65

EXCLUSION(S):
All Medicare Assessments coded in AA8B
Admission Assessment at the Target Assessment
Residents satisfying any of the following conditions:
H3d is missing on either the target assessment or the prior assessment.
The resident has end-stage disease (J5c = checked) or status is unknown (J5c = missing) on the target assessment.
The resident is receiving hospice care (P1ao = checked) or hospice status is unknown (P1ao = missing) on the target assessment.

STRATIFICATION:
CMI

27 CNT01 – Percent of Residents who are bladder or bowel incontinent

NUMERATOR:
Residents who were frequently incontinent or fully incontinent on target assessment (H1a=3 or 4, or H1b=3 or 4).

DENOMINATOR:
All residents with a valid target assessment.

COVARIATE(S):
PSI: Subset 1 Diagnoses
PSI: Subset 2 Non-Diagnoses
RUG Late Loss ADL Scale
Age less than 65
RUG Nursing CMI

EXCLUSION(S):
All Medicare Assessments coded in AA8B
Admission Assessment at the Target Assessment
Residents satisfying any of the following conditions are excluded
The resident has end-stage disease (J5c = checked) or status is unknown (J5c = missing) on the target assessment.
The resident is receiving hospice care (P1ao = checked) or hospice status is unknown (P1ao = missing) on the target assessment.

STRATIFICATION:
ADL-Long

28 CNT02 – Percent of Residents with worsening bowel continence

NUMERATOR:
Residents with a value for bowel incontinence greater at target assessment relative to prior assessment (H1a[t]>H1a[t-1]).

DENOMINATOR:
All residents with a valid target assessment and a valid prior assessment.

COVARIATE(S):
RUG Nursing CMI
PSI: Subset 1 Diagnoses
PSI: Subset 2 Non-Diagnoses
Age less than 65

EXCLUSION(S):
All Medicare Assessments coded in AA8B
Two Consecutive Chronic Admission Assessments
The value of H1a on the prior assessment is the maximum value (H1a[t-1] = 4).
The H1a value is missing on the target assessment [t].
The H1a value is missing on the prior assessment [t-1] and the H1a value shows some impairment on the target assessment (H1a[t]> 0).
The resident is comatose (B1 = 1) or comatose status is unknown (B1 = missing) on the target assessment.
The resident has end-stage disease (J5c = checked) or status is unknown (J5c = missing) on the target assessment.
The resident is receiving hospice care (P1ao = checked) or hospice status is unknown (P1ao = missing) on the target assessment.
The resident has an ostomy present (H3i checked) or ostomy status is unknown (H3i = missing) on the target assessment.

STRATIFICATION:
ADL-Long

29 CNT03 – Percent of Residents with worsening bladder continence

NUMERATOR:
Residents with a value for bladder incontinence greater at target assessment relative to prior assessment (H1b[t]>H1b[t-1]).

DENOMINATOR:
All residents with a valid target assessment and a valid prior assessment.

COVARIATE(S):
PSI: Subset 1 Diagnoses
PSI: Subset 2 Non-Diagnoses
Cognitive Performance Scale
RUG Nursing CMI
Age less than 65

EXCLUSION(S):
All Medicare Assessments coded in AA8B
Two Consecutive Chronic Admission Assessments
The value of H1b on the prior assessment is the maximum value (H1b[t-1] = 4).
The H1b value is missing on the target assessment [t].
The H1b value is missing on the prior assessment [t-1] and the H1b value shows some impairment on the target assessment (H1b[t]> 0).
The resident is comatose (B1 = 1) or comatose status is unknown (B1 = missing) on the target assessment.
The resident has end-stage disease (J5c = checked) or status is unknown (J5c = missing) on the target assessment.
The resident is receiving hospice care (P1ao = checked) or hospice status is unknown (P1ao = missing) on the target assessment.

STRATIFICATION:
ADL-Long

30 CNT04 – Percent of Residents with a urinary tract infection

NUMERATOR:
Residents with urinary tract infection on target assessment (I2j = checked).

DENOMINATOR:
All residents with a valid target assessment.

COVARIATE(S):
Age less than 65

EXCLUSION(S):
All Medicare Assessments coded in AA8B
Admission Assessment at the Target Assessment
Residents satisfying any of the following conditions:
I2j is missing on the target assessment.
The resident has end-stage disease (J5c = checked) or status is unknown (J5c = missing) on the target assessment.
The resident is receiving hospice care (P1ao = checked) or hospice status is unknown (P1ao = missing) on the target assessment.

STRATIFICATION:
CMI

31 CNT2A – Percent of Residents with improving bowel continence

NUMERATOR:
Residents with a value for bowel incontinence less at target assessment relative to prior assessment (H1a[t]<H1a[t-1]).

DENOMINATOR:
All residents with a valid target assessment and a valid prior assessment.

COVARIATE(S):
Age less than 65
PSI: Subset 1 Diagnoses

EXCLUSION(S):
All Medicare Assessments coded in AA8B
Two Consecutive Chronic Admission Assessments
The value of H1a on the prior assessment is the mimimum value (H1a[t-1] = 0)
The H1a value is missing on the target assessment [t].
The H1a value is missing on the prior assessment [t-1] and the H1a value shows some impairment on the target assessment (H1a[t]> 0)
The resident is comatose (B1 = 1) or comatose status is unknown (B1 = missing) on the target assessment.
The resident has end-stage disease (J5c = checked) or status is unknown (J5c = missing) on the target assessment.
The resident is receiving hospice care (P1ao = checked) or hospice status is unknown (P1ao = missing) on the target assessment.
The resident has an ostomy present (H3i checked) or ostomy status is unknown (H3i = missing) on the target assessment.

STRATIFICATION:
CPS

32 CNT3A – Percent of Residents with improving bladder continence

NUMERATOR:
Residents with a value for bladder incontinence less at target assessment relative to prior assessment (H1b[t]<H1b[t-1]).

DENOMINATOR:
All residents with a valid target assessment and a valid prior assessment.

COVARIATE(S):
Age less than 65
PSI: Subset 1 Diagnoses

EXCLUSION(S):
All Medicare Assessments coded in AA8B
Two Consecutive Chronic Admission Assessments
The value of H1b on the prior assessment is the mimimum value (H1b[t-1] = 0)
The H1b value is missing on the target assessment [t].
The H1b value is missing on the prior assessment [t-1] and the H1b value shows some impairment on the target assessment (H1b[t]> 0)
The resident is comatose (B1 = 1) or comatose status is unknown (B1 = missing) on the target assessment.
The resident has end-stage disease (J5c = checked) or status is unknown (J5c = missing)
The resident is receiving hospice care (P1ao = checked) or hospice status is unknown (P1ao = missing) on the target assessment.

STRATIFICATION:
CPS

33 CNTD2 – Percent of Residents who have declined bowel continence

NUMERATOR:
Residents with a value for bowel incontinence greater at target assessment relative to prior assessment (H1a[t]>H1a[t-1]).

DENOMINATOR:
All residents with a valid target assessment and a valid prior assessment.

COVARIATE(S):
RUG Nursing CMI
PSI: Subset 1 Diagnoses
PSI: Subset 2 Non-Diagnoses
Age less than 65

EXCLUSION(S):
All Medicare Assessments coded in AA8B
Two Consecutive Chronic Admission Assessments
The resident is comatose (B1=1,yes)
The resident has end-stage disease (J5c = checked)
Hospice (P1ao = checked)
The resident has an ostomy present (H3i checked)

STRATIFICATION:
ADL-Long

34 CNTD3 – Percent of Residents who have declined bladder continence

NUMERATOR:
Residents with a value for bladder incontinence greater at target assessment relative to prior assessment (H1b[t]>H1b[t-1]).

DENOMINATOR:
All residents with a valid target assessment and a valid prior assessment.

COVARIATE(S):
PSI: Subset 1 Diagnoses
PSI: Subset 2 Non-Diagnoses
Cognitive Performance Scale
RUG Nursing CMI
Age less than 65

EXCLUSION(S):
All Medicare Assessments coded in AA8B
Two Consecutive Chronic Admission Assessments
The resident is comatose (B1=1,yes)
The resident has end-stage disease (J5c = checked)
Hospice (P1ao = checked)

STRATIFICATION:
CPS

35 CNTI2 – Percent of Residents who have improved bowel continence

NUMERATOR:
Residents with a value for bowel incontinence less at target assessment relative to prior assessment (H1a[t]<H1a[t-1]).

DENOMINATOR:
All residents with a valid target assessment and a valid prior assessment.

COVARIATE(S):
PSI: Subset 1 Diagnoses
Age less than 65

EXCLUSION(S):
All Medicare Assessments coded in AA8B
Two Consecutive Chronic Admission Assessments
The resident is comatose (B1=1,yes)
The resident has end-stage disease (J5c = checked)
Hospice (P1ao = checked)
The resident has an ostomy present (H3i checked)

STRATIFICATION:
CPS

36 CNTI3 – Percent of Residents who have improved bladder continence

NUMERATOR:
Residents with a value for bladder incontinence less at target assessment relative to prior assessment (H1b[t]<H1b[t-1]).

DENOMINATOR:
All residents with a valid target assessment and a valid prior assessment.

COVARIATE(S):
PSI: Subset 1 Diagnoses
Age less than 65

EXCLUSION(S):
All Medicare Assessments coded in AA8B
Two Consecutive Chronic Admission Assessments
The resident is comatose (B1=1,yes)
The resident has end-stage disease (J5c = checked)
Hospice (P1ao = checked)

STRATIFICATION:
CPS

37 CNTX2 – Percent of Residents who failed to improve their bladder incontinence

NUMERATOR:
1. Residents who did not have a catheter (H3d[t-1] not checked (value 0)) on the prior assessment AND was fully bladder continent (H1b[t-1] = 0) AND who had a catheter (H3d[t] checked (value 1)) OR was less than fully bladder continent (H1b[t] >0) on the Target Assessment.
Residents who have a catheter (H3d[t-1] checked (value 1)) on the prior assessmwas bladder incontinent (H1b[t-1] = 1) AND
2. Residents who did not have a catheter (H3d[t-1] not checked (value 0)) on the prior assessment AND was worse bladder continent (H1b[t-1] gt 0) AND who had a catheter (H3d[t] checked (value 1)) OR same or worse on bladder continent (H1b[t] >=H1b[t-1]))
3. Residents who did have a catheter (H3d[t-1] checked (value 1)) on the prior assessment AND
who did have a catheter (H3d[t] checked (value 1)) and was frequently or fully incontinent (H1b[t] >2) .

DENOMINATOR:
All residents with a valid target assessment and a valid prior assessment.

COVARIATE(S):
RUG Nursing CMI
Age less than 65
Cognitive Performance Scale

EXCLUSION(S):
All Medicare Assessments coded in AA8B
Two Consecutive Chronic Admission Assessments
Residents satisfying the following condition:
There are missing values for H1b or H3d on either the Prior or Target assessment.
The resident is comatose (B1 = 1) or comatose status is unknown (B1= missing) on the Target assessment.
The resident has paraplegia (I1x = 1) or paraplegia status unknown (I1x missing) on the Target assessment.
The resident has quadriplegia (I1z = 1) or quadriplegia status unknown (I1z missing) on the Target assessment.

STRATIFICATION:
ADL-Long

38 COG01 – Percent of Residents whose cognitive ability has worsened

NUMERATOR:
Residents with score on cognitive performance scale (CPS, Morris et al. 1994) that is higher on target relative to prior assessment (CPS[t]>CPS[t-1]).

.

DENOMINATOR:
All residents with a valid target assessment and a valid prior assessment.

COVARIATE(S):
Age less than 65

EXCLUSION(S):
All Medicare Assessments coded in AA8B
Two Consecutive Chronic Admission Assessments
The CPS score has a missing value on the target assessment [t].
The CPS score has a missing value on the prior assessment [t-1] and the CPS score shows some impairment on the target assessment (CPS[t] $>$ 0).
The CPS score on the prior assessment [t-1] is at the maximum value of 6.
The resident is comatose (B1 = 1) or comatose status is unknown (B1= missing) on the target assessment.
The resident has end-stage disease (J5c = checked) or status is unknown (J5c = missing) on the target assessment.
The resident is receiving hospice care (P1ao = checked) or hospice status is unknown (P1ao = missing) on the target assessment.

STRATIFICATION:
CMI

39 COG1A – Percent of Residents whose cognitive ability has improved

NUMERATOR:
Residents with score on cognitive performance scale (CPS, Morris et al. 1994) that is lower on target relative to prior assessment (CPS[t]<CPS[t-1]).

DENOMINATOR:
All residents with a valid target assessment and a valid prior assessment.

COVARIATE(S):
Age less than 65
Full PSI
PSI: Subset 1 Diagnoses

EXCLUSION(S):
All Medicare Assessments coded in AA8B
Two Consecutive Chronic Admission Assessments
The CPS score has a missing value on the target assessment
The CPS score has a missing value on the prior assessment [t-1] and the CPS score shows some impairment on the target assessment (CPS[t] > 0).
The CPS score on the prior assessment [t-1] is at the muinimum value of 0.
The resident is comatose (B1 = 1) or comatose status is unknown (B1= missing) on the target assessment.
The resident has end-stage disease (J5c = checked) or status is unknown (J5c = missing) on the target assessment.
The resident is receiving hospice care (P1ao = checked) or hospice status is unknown (P1ao = missing) on the target assessment.

STRATIFICATION:
CPS

40 COGD1 – Percent of Residents whose ability to communicate has declined

NUMERATOR:
Residents with score on cognitive performance scale (CPS, Morris et al. 1994) that is higher on target relative to prior assessment (CPS[t]>CPS[t-1]).

DENOMINATOR:
All residents with a valid target assessment and a valid prior assessment.

COVARIATE(S):
Age less than 65

EXCLUSION(S):
All Medicare Assessments coded in AA8B
Two Consecutive Chronic Admission Assessments
The resident is comatose (B1=1,yes)
The resident has end-stage disease (J5c = checked)
Hospice (P1ao = checked)

STRATIFICATION:
ADL-Long

41 COGI1 – Percent of Residents who have improved cognitive performance

NUMERATOR:
Residents with score on cognitive performance scale (CPS, Morris et al. 1994) that is lower on target relative to prior assessment (CPS[t]<CPS[t-1]).

DENOMINATOR:
All residents with a valid target assessment and a valid prior assessment.

COVARIATE(S):
Full PSI
PSI: Subset 1 - Diagnoses
Age less than 65

EXCLUSION(S):
All Medicare Assessments coded in AA8B
Two Consecutive Chronic Admission Assessments
The resident is comatose (B1=1,yes)
The resident has end-stage disease (J5c = checked)
Hospice (P1ao = checked)

STRATIFICATION:
CPS

42 COM01 – Percent of Residents whose ability to communicate has worsened

NUMERATOR:
Residents with a Communication Scale score (sum of 'ability to understand others' (C6) and 'making self understood' (C4)) that is greater at the target assessment relative to the prior assessment (C4[t]+C6[t] > C4[t-1]+C6[t-1]).

DENOMINATOR:
All residents with a valid target assessment and a valid prior assessment.

COVARIATE(S):
Short term memory problem
Long term memory problem
Age less than 65

EXCLUSION(S):
All Medicare Assessments coded in AA8B
Two Consecutive Chronic Admission Assessments
The Communication Scale score is missing on the target assessment [t].
The Communication Scale score is missing on the prior assessment [t-1] and the Communication Scale score shows some impairment on the target assessment (Communication Scale[t] > 0).
The Communication Scale score on the prior assessment [t-1] is at the maximum value of 6.
The resident is comatose (B1 = 1) or comatose status is unknown (B1= missing) on the target assessment.
The resident has end-stage disease (J5c = checked) or status is unknown (J5c = missing) on the target assessment.
The resident is receiving hospice care (P1ao = checked) or hospice status is unknown (P1ao = missing) on the target assessment.

STRATIFICATION:
CPS

43 COM1A – Percent of Residents whose ability to communicate has improved

NUMERATOR:
Residents with a Communication Scale score (sum of 'ability to understand others' (C6) and 'making self understood' (C4)) that is lower at the target assessment relative to the prior assessment (C4[t]+C6[t] < C4[t-1]+C6[t-1]).

DENOMINATOR:
All residents with a valid target assessment and a valid prior assessment.

COVARIATE(S):
Age less than 65
PSI: Subset 1 Diagnoses

EXCLUSION(S):
All Medicare Assessments coded in AA8B
Two Consecutive Chronic Admission Assessments
The Communication Scale score is missing on the target assessment [t].
The Communication Scale score is missing on the prior assessment [t-1] and the Communication Scale score shows some impairment on the target assessment (Communication Scale[t] > 0).
The Communication Scale score on the prior assessment [t-1] is at the minimum value of 0.
The resident is comatose (B1 = 1) or comatose status is unknown (B1= missing) on the target assessment.
The resident has end-stage disease (J5c = checked) or status is unknown (J5c = missing) on the target assessment
The resident is receiving hospice care (P1ao = checked) or hospice status is unknown (P1ao = missing) on the target assessment.

STRATIFICATION:
CPS

44 COMD1 – Percent of Residents whose ability to communicate has worsened

NUMERATOR:
Residents with a Communication Scale score (sum of 'ability to understand others' (C6) and 'making self understood' (C4)) that is greater at the target assessment relative to the prior assessment (C4[t]+C6[t] > C4[t-1]+C6[t-1]).

DENOMINATOR:
All residents with a valid target assessment and a valid prior assessment.

COVARIATE(S):
Short term memory problem
Long term memory problem
Age less than 65

EXCLUSION(S):
All Medicare Assessments coded in AA8B
Two Consecutive Chronic Admission Assessments
The resident is comatose (B1=1,yes)
The resident has end-stage disease (J5c = checked)
Hospice (P1ao = checked)

STRATIFICATION:
CPS

45 COMI1 – Percent of Residents who have improved communication

NUMERATOR:
Residents with a Communication Scale score (sum of 'ability to understand others' (C6) and 'making self understood' (C4)) that is lower at the target assessment relative to the prior assessment (C4[t]+C6[t]
< C4[t-1]+C6[t-1]).

DENOMINATOR:
All residents with a valid target assessment and a valid prior assessment.

COVARIATE(S):
PSI: Subset 1 - Diagnoses
Age less than 65

EXCLUSION(S):
All Medicare Assessments coded in AA8B
Two Consecutive Chronic Admission Assessments
The resident is comatose (B1=1,yes)
The resident has end-stage disease (J5c = checked)
Hospice (P1ao = checked)

STRATIFICATION:
CPS

46 DEL0X – Percent of Residents with symptoms of delirium

NUMERATOR:
Residents satisfying any of the following 3 conditions: 1. Any delirium symptom that departs from usual functioning (B5a[t] through B5f[t] = 2) on target assessment.
2. Any delirium symptom (B5a through B5f) that departs from usual functioning on the prior assessment AND is present on the target assessment (e.g., for B5a: B5a[t-1] = 2 and B5a[t] = 1).
3. Any delirium symptom (B5a through B5f) that was not present on the prior assessment AND is present on the target assessment (e.g., for B5a: B5a[t-1] = 0 and B5a[t] =1)
AND the Cognitive Performance Scale (CPS) score indicates that cognitive impairment is not severe (CPS = 0, 1, 2, or 3) on the target assessment.

DENOMINATOR:
All residents with a valid target assessment and a valid prior assessment.

COVARIATE(S):
Age less than 65

EXCLUSION(S):
All Medicare Assessments coded in AA8B
Two Consecutive Chronic Admission Assessments
There is a missing value on any of the items B5a through B5f on the target assessment [t].
For items B5a through B5f, there is missing data on the prior assessment [t-1] the value for the same item on the target assessment [t] is > 0 showing delirium.
The resident is comatose (B1 = 1) or comatose status is unknown (B1= missing) on the target assessment.
The resident has end-stage disease (J5c = checked) or status is unknown (J5c = missing) on the target assessment.
The resident is receiving hospice care (P1ao = checked) or hospice status is unknown (P1ao = missing) on the target assessment.

STRATIFICATION:
DRS

47 DRG01 – Percent of Residents on antipsychotics without a diagnosis of psychosis

NUMERATOR:
Residents receiving antipsychotics (O4a>0) on target assessment.

DENOMINATOR:
All residents with a valid target assessment.

COVARIATE(S):
Motor Agitation
Moderate/impaired decision making problem
Long term memory problem
Cognitive Performance Scale
Combination Alzheimers Disease/Other Dementia
Age less than 65

EXCLUSION(S):
All Medicare Assessments coded in AA8B
Admission Assessment at the Target Assessment
Residents satisfying any of the following conditions are excluded:
A psychiatric disorder is indicated by ICD9 diagnosis code on the target assessment or most recent full assessment: I3a through I3e = 295.00-295.95 or 297.00-298.9.
Schizophrenia is indicated on the target assessment or most recent full assessment by I1gg = checked or the value of I1gg is missing,
Tourette syndrome is indicated by ICD9 diagnosis code on the target assessment or most recent full assessment: I3a through I3e=307.23.
Huntington's syndrome is indicated by ICD9 diagnosis code on the target assessment or most recent full assessment: I3a through I3e=333.4.
Hallucinations are present on the target assessment only: J1i = checked (value 1) or the value of J1i is missing.
The value of O4a is missing on the target assessment.
9. Resident has end-stage disease (J5c = checked) or the status of end-stage disease is unknown (J5c = missing) on the target assessment.
The resident is receiving hospice care (P1ao = checked) or hospice status is unknown (P1ao = missing) on the target assessment.

STRATIFICATION:
CMI

48 FAL01 – Percent of Residents who have fallen

NUMERATOR:
Residents who had a fall in the last 30 days recorded on the target assessment (J4a[t]=checked).

DENOMINATOR:
All residents with a valid target assessment and a valid prior assessment.

COVARIATE(S):
Locomotion Problem
Not totally dependent in transferring
Unsteady gait/cognitive impairment
Any Wandering
Age less than 65

EXCLUSION(S):
All Medicare Assessments coded in AA8B
Two Consecutive Chronic Admission Assessments
The prior assessment indicates a fall in the last 30 days (J4a[t-1] = checked).
The value of J4a is missing on the target assessment [t] or prior assessment [t-1].

STRATIFICATION:
CMI

49 FAL02 – Percent of Residents who have fallen in the last 30-days

NUMERATOR:
All residents who had a fall in the last 30 days recorded on the target assessment (J4a[t]=checked).

DENOMINATOR:
All residents with a valid target assessment.

COVARIATE(S):
Not totally dependent in transferring
Locomotion Problem
PSI: Subset 2 Non-Diagnoses
Any Wandering
Unsteady gait/cognitive impairment
Age less than 65

EXCLUSION(S):
All Medicare Assessments coded in AA8B
Admission Assessment at the Target Assessment
The value of J4a is missing on the target assessment [t]

STRATIFICATION:
CMI

50 INF0X – Percent of Residents with infections

NUMERATOR:
Residents with any of the following infections or health conditions noted on the target assessment.
1. Pneumonia (I2e=checked) on the target assessment
2. Respiratory infection (I2f=checked) on the target assessment
3. Septicemia (I2g=checked) on the target assessment
4. Urinary tract infection (I2j=checked) on the target assessment only,
5. Viral hepatitis (I2k=checked) on the target assessment
6. Wound infection (I2l=checked) on the target assessment
7. Fever (J1h=checked) on the target assessment
8. Recurrent lung aspiration (J1k=checked) on the target assessment

DENOMINATOR:
All residents with a valid target assessment

COVARIATE(S):
Age less than 65

EXCLUSION(S):
All Medicare Assessments coded in AA8B
Admission Assessment at the Target Assessment
Residents satisfying any of the following conditions:
The the value of any of the infections and/or health conditions (I2e, I2f, I2g, I2j, I2k, I2l, J1h, or J1k) selected from the target assessment is missing.

The resident has end-stage disease (J5c = checked) or status is unknown (J5c = missing) on the target assessment.
The resident is receiving hospice care (P1ao = checked) or hospice status is unknown (P1ao = missing) on the target assessment.

STRATIFICATION:
CMI

51 MOB01 – Percent of Residents who have declined in their ability to locomote

NUMERATOR:
Total number of residents whose value locomotion self-performance is greater at target relative to prior assessment (G1e(A)[t]>G1e(A)[t-1]). Recode (8=4)

DENOMINATOR:
All residents with a valid target assessment and a valid prior assessment.

COVARIATE(S):
PSI: Subset 1 Diagnoses
More dependence in toileting
Requires much assistance for eating
Age less than 65

EXCLUSION(S):
All Medicare Assessments coded in AA8B
Two Consecutive Chronic Admission Assessments
The G1e(A) value is missing on the target assessment [t].
The G1e(A) value is missing on the prior assessment [t-1] and the G1e(A) value shows some dependence on the target assessment (G1e(A)[t]>0).
The G1e(A) value on the prior assessment is 4 (total dependence) or 8 (activity did not occur).
The resident is comatose (B1 = 1) or comatose status is unknown (B1 = missing) on the target assessment.
The resident has end-stage disease (J5c = checked) or status is unknown (J5c = missing) on the target assessment.
The resident is receiving hospice care (P1ao = checked) or hospice status is unknown (P1ao = missing) on the target assessment.

STRATIFICATION:
CMI

52 MOB1A – Percent of Residents who have improved in their ability to locomote

NUMERATOR:
Total number of residents whose value locomotion self-performance is less at target relative to prior assessment (G1e(A)[t]<G1e(A)[t-1]). Recode (8=4)

DENOMINATOR:
All residents with a valid target assessment and a valid prior assessment.

COVARIATE(S):
Age less than 65
PSI: Subset 2 Non-Diagnoses
Cognitive Performance Scale
Requires much assistance for eating

EXCLUSION(S):
All Medicare Assessments coded in AA8B
Two Consecutive Chronic Admission Assessments
The G1e(A) value is missing on the target assessment [t].
The G1e(A) value is missing on the prior assessment [t-1] and the G1e(A) value shows some dependence on the target assessment (G1e(A)[t]>0).
The G1e(A) value on the prior assessment is 0 (independence)
The resident is comatose (B1 = 1) or comatose status is unknown (B1 = missing) on the target assessment.
The resident has end-stage disease (J5c = checked) or status is unknown (J5c = missing) on the target assessment.
The resident is receiving hospice care (P1ao = checked) or hospice status is unknown (P1ao = missing) on the target assessment.

STRATIFICATION:
CPS

53 MOBD1 – Percent of Residents who have declined in their ability to locomote

NUMERATOR:
Total number of residents whose value locomotion self-performance is greater at target relative to prior assessment (G1e(A)[t]>G1e(A)[t-1]). Recode (8=4)

DENOMINATOR:
All residents with a valid target assessment and a valid prior assessment.

COVARIATE(S):
Not totally dependent in transferring
Locomotion Problem
PSI: Subset 2 Non-Diagnoses
Age less than 65

EXCLUSION(S):
All Medicare Assessments coded in AA8B
Two Consecutive Chronic Admission Assessments
The resident is comatose (B1=1,yes)
The resident has end-stage disease (J5c = checked)
Hospice (P1ao = checked)

STRATIFICATION:
CMI

54 MOBI1 – Percent of Residents who have improved in their ability to locomote

NUMERATOR:
Total number of residents whose value locomotion self-performance is less at target relative to prior assessment (G1e(A)[t]<G1e(A)[t-1]). Recode (8=4)

DENOMINATOR:
All residents with a valid target assessment and a valid prior assessment.

COVARIATE(S):
PSI: Subset 2 - Non-Diagnoses
Cognitive Performance Scale
Requires much assistance for eating
Age less than 65

EXCLUSION(S):
All Medicare Assessments coded in AA8B
Two Consecutive Chronic Admission Assessments
The resident is comatose (B1=1,yes)
The resident has end-stage disease (J5c = checked)
Hospice (P1ao = checked)

STRATIFICATION:
CPS

55 MOD03 – Percent of Residents who have become more depressed or anxious

NUMERATOR:
The total number of residents whose Mood Scale score is greater on target assessment relative to prior assessment (Mood Scale [t] > Mood Scale [t-1].

DENOMINATOR:
All residents with a valid target assessment and a valid prior assessment.

COVARIATE(S):
Age less than 65

EXCLUSION(S):
All Medicare Assessments coded in AA8B
Two Consecutive Chronic Admission Assessments
The Mood Scale score is missing on the target assessment [t].
The Mood Scale score is missing on the prior assessment [t-1] and the Mood Scale score indicates symptoms present on the target assessment (Mood Scale[t] > 0).
The Mood Scale score is at a maximum (value 8) on the prior assessment.
The resident is comatose (B1=1) or comatose status is unknown (B1=missing) on the target assessment.

STRATIFICATION:
DRS

56 MOD04 – Percent of Residents who improve their mood or remain free from symptoms of depression (based on MDS Depression Rating Scale)

NUMERATOR:
Residents who have a DRS scale change score that is negative between the Prior and Target assessments (DRS[t]-DRS[t-1]<0) OR
Residents who have a DRS score of 0 at Prior AND Target assessments.

DENOMINATOR:
All residents with a valid target assessment and a valid prior assessment.

COVARIATE(S):
Age less than 65

EXCLUSION(S):
All Medicare Assessments coded in AA8B
Two Consecutive Chronic Admission Assessments
Residents with non-valid DRS at the Target assessment (DRS[t] = missing)
Residents with non-valid DRS at the Prior assessment (DRS[t-1]=missing) AND DRS greater than 0 at the Target assessment (DRS[t]>0).

STRATIFICATION:
CMI

57 MOD4A – Percent of Residents who decline in mood from symptoms of depression (based on MDS Depression Rating Scale)

NUMERATOR:
Residents who have a DRS scale change score that is positive between the Prior and Target assessments (DRS[t]-DRS[t-1]>0)

DENOMINATOR:
All residents with a valid target assessment and a valid prior assessment.

COVARIATE(S):
Age less than 65

EXCLUSION(S):
All Medicare Assessments coded in AA8B
Two Consecutive Chronic Admission Assessments
Residents with non-valid DRS at the Target assessment (DRS[t] = missing)
Residents with non-valid DRS at the Prior assessment (DRS[t-1]=missing) AND DRS greater than 0 at the Target assessment (DRS[t]>0).
Residents with maximum DRS (value 14) on prior assessment

STRATIFICATION:
CMI

58 MODD4 – Percent of Residents who decline in mood from symptoms of depression (based on MDS Depression Rating Scale)

NUMERATOR:
Residents who have a DRS scale change score that is positive between the Prior and Target assessments (DRS[t]-DRS[t-1]>0)

DENOMINATOR:
All residents with a valid target assessment and a valid prior assessment.

COVARIATE(S):
Age less than 65

EXCLUSION(S):
All Medicare Assessments coded in AA8B
Two Consecutive Chronic Admission Assessments

STRATIFICATION:
CMI

59 MODI4 – Percent of Residents who improved in mood from symptoms of depression (based on MDS Depression Rating Scale)

NUMERATOR:
Residents who have a DRS scale change score that is negative between the Prior and Target assessments (DRS[t]-DRS[t-1]<0)

DENOMINATOR:
All residents with a valid target assessment and a valid prior assessment.

COVARIATE(S):
Age less than 65

EXCLUSION(S):
All Medicare Assessments coded in AA8B
Two Consecutive Chronic Admission Assessments

STRATIFICATION:
CPS

60 NUT01 – Percent of Residents with a feeding tube

NUMERATOR:
All residents with a feeding tube at target assessment (K5b=checked).

DENOMINATOR:
All residents with a valid target assessment.

COVARIATE(S):
RUG Clinically Complex
Swallowing problem
RUG Nursing CMI
Age less than 65

EXCLUSION(S):
All Medicare Assessments coded in AA8B
Admission Assessment at the Target Assessment
Residents satisfying any of the following conditions:
K5b is missing on the target assessment.
The resident is comatose (B1 = 1) or comatose status is unknown (B1 = missing) on the target assessment.
The resident has end-stage disease (J5c = checked) or status is unknown (J5c = missing) on the target assessment.
The resident is receiving hospice care (P1ao = checked) or hospice status is unknown (P1ao = missing) on the target assessment.

STRATIFICATION:
ADL-Long

61 PAI0X – Percent of Residents with pain

NUMERATOR:
Residents with moderate pain at least daily (J2a=2 AND J2b=2) OR horrible/excruciating pain at any frequency (J2b=3) on the target assessment.

DENOMINATOR:
All residents with a valid target assessment.

COVARIATE(S):
Cognitive Performance Scale
Long term memory problem
Age less than 65

EXCLUSION(S):
All Medicare Assessments coded in AA8B
Admission Assessment at the Target Assessment
Residents satisfying any of the following conditions:
Either J2a or J2b is missing on the target assessment.
The values of J2a and J2b are inconsistent on the target assessment.

STRATIFICATION:
DRS

62 PAN01 – Percent of Residents with worsening pain

NUMERATOR:
Residents with greater pain at target assessment relative to prior assessment, defined by greater score on the Pain Scale.

DENOMINATOR:
All residents with a valid target assessment and a valid prior assessment.

COVARIATE(S):
Age less than 65

EXCLUSION(S):
All Medicare Assessments coded in AA8B
Two Consecutive Chronic Admission Assessments
The Pain Scale value is missing on the target assessment [t].
The Pain Scale value is missing on the prior assessment [t-1] and the Pain Scale value shows presence of pain on the target assessment (Pain Scale[t]$>$ 0).
The Pain Scale score is a maximum (value 3) on the prior assessment.

STRATIFICATION:
CMI

63 PAN1A – Percent of Residents with improving pain

NUMERATOR:
Residents with less pain at target assessment relative to prior assessment, defined by greater score on the Pain Scale.

DENOMINATOR:
All residents with a valid target assessment and a valid prior assessment.

COVARIATE(S):
Age less than 65
Cognitive Performance Scale
PSI: Subset 1 Diagnoses

EXCLUSION(S):
All Medicare Assessments coded in AA8B
Two Consecutive Chronic Admission Assessments
The Pain Scale value is missing on the target assessment [t].
The Pain Scale value is missing on the prior assessment [t-1] and the Pain Scale value shows presence of pain on the target assessment (Pain Scale[t]$>$ 0).
The Pain Scale score is a minimum (value 0) on the prior assessment.

STRATIFICATION:
CPS

64 PAND1 – Percent of Residents who have declined in pain

NUMERATOR:
Residents with greater pain at target assessment relative to prior assessment, defined by greater score on the Pain Scale.

DENOMINATOR:
All residents with a valid target assessment and a valid prior assessment.

COVARIATE(S):
Age less than 65

EXCLUSION(S):
All Medicare Assessments coded in AA8B
Two Consecutive Chronic Admission Assessments

STRATIFICATION:
CPS

65 PANI1 – Percent of Residents who have improved pain

NUMERATOR:
Residents with less pain at target assessment relative to prior assessment, defined by greater score on the Pain Scale.

DENOMINATOR:
All residents with a valid target assessment and a valid prior assessment.

COVARIATE(S):
PSI: Subset 1 - Diagnoses
Cognitive Performance Scale
Age less than 65

EXCLUSION(S):
All Medicare Assessments coded in AA8B
Two Consecutive Chronic Admission Assessments

STRATIFICATION:
CPS

66 PRU01 – Percent of Residents with pressure sores

NUMERATOR:
Residents with pressure ulcers (Stage 1-4) on target assessment (M2a >0 OR I3a-e = 707.0)

DENOMINATOR:
All residents with a valid target assessment.

COVARIATE(S):
PSI: Subset 1 Diagnoses
PSI: Subset 2 Non-Diagnoses
Age less than 65

EXCLUSION(S):
All Medicare Assessments coded in AA8B
Admission Assessment at the Target Assessment
Residents satisfying any of the following conditions are excluded
The value of M2a is missing on the target assessment.

STRATIFICATION:
CMI

67 PRU04 – Percent of Residents with worsening pressure sores

NUMERATOR:
Total number of residents evidencing more severe pressure ulcers on the target assessment versus the prior assessment (M2a[t] is greater than M2a[t-1]).

DENOMINATOR:
All residents with a valid target assessment and a valid prior assessment.

COVARIATE(S):
RUG Late Loss ADL
Age less than 65

EXCLUSION(S):
All Medicare Assessments coded in AA8B
Two Consecutive Chronic Admission Assessments
Residents satisfying any of the following conditions:
The M2a value is missing on the target assessment [t].
The M2a value is missing on the prior assessment [t-1] and the M2a value shows presence of ulcers
on the target assessment (M2a[t]> 0).

STRATIFICATION:
CMI

68 PRU05 – Percent of Residents who have a Pressure Ulcer Stage 2 to 4

NUMERATOR:
All residents who had a stage 2 to 4 pressure ulcer on target assessment [m2a gt 1]

DENOMINATOR:
All residents with a valid target assessment

COVARIATE(S):
RUG Cognitive Impairment
PSI: Subset 1 Diagnoses
More dependence in toileting
Age less than 65

EXCLUSION(S):
All Medicare Assessments coded in AA8B
Admission Assessment at the Target Assessment

STRATIFICATION:
CMI

69 PRU09 – Percent of Residents who have a newly ocurring pressure ulcer stage 2 to 4

NUMERATOR:
All residents who had a stage 2 to 4 pressure ulcer on target assessment [m2a gt 1], and no stage 2 to 4 on prior assessment [m2a(t-1)<2]

DENOMINATOR:
All residents with a valid target assessment and a valid prior assessment.

COVARIATE(S):
Age less than 65
PSI: Subset 1 Diagnoses
More dependence in toileting
RUG Cognitive Impairment

EXCLUSION(S):
All Medicare Assessments coded in AA8B
Two Consecutive Chronic Admission Assessments
The M2a value is missing on the target assessment [t].
The value of M2a on the prior assessment is ge 2.

STRATIFICATION:
CMI

70 PRU4A – Percent of Residents with improving pressure sores

NUMERATOR:
Total number of residents evidencing less severe pressure ulcers on the target assessment versus the prior assessment (M2a[t] is less than M2a[t-1]).

DENOMINATOR:
All residents with a valid target assessment and a valid prior assessment.

COVARIATE(S):
RUG Cognitive Impairment
Full PSI
PSI: Subset 2 - Non-Diagnoses
More dependence in toileting
Locomotion Problem
Age less than 65

EXCLUSION(S):
All Medicare Assessments coded in AA8B
Two Consecutive Chronic Admission Assessments
The M2a value is missing on the target assessment [t].
The M2a value is missing on the prior assessment [t-1] and the M2a value shows presence of ulcers
on the target assessment (M2a[t]> 0).

STRATIFICATION:
CPS

71 PRUD4 – Percent of Residents who have declined pressure ulcer

NUMERATOR:
Total number of residents evidencing more severe pressure ulcers on the target assessment versus the prior assessment (M2a[t] is greater than M2a[t-1]).

DENOMINATOR:
All residents with a valid target assessment and a valid prior assessment.

COVARIATE(S):
RUG Late Loss ADL
Age less than 65

EXCLUSION(S):
All Medicare Assessments coded in AA8B
Two Consecutive Chronic Admission Assessments

STRATIFICATION:
ADL-Long

72 PRUI4 – Percent of Residents who have improved pressure ulcer

NUMERATOR:
Total number of residents evidencing less severe pressure ulcers on the target assessment versus the prior assessment (M2a[t] is less than M2a[t-1]).

DENOMINATOR:
All residents with a valid target assessment and a valid prior assessment.

COVARIATE(S):
RUG Cognitive Impairment
Full PSI
PSI: Subset 2 - Non-Diagnoses
More dependence in toileting
Locomotion Problem
Age less than 65

EXCLUSION(S):
All Medicare Assessments coded in AA8B
Two Consecutive Chronic Admission Assessments

STRATIFICATION:
CPS

73 RES01 – Percent of Residents in physical restraints

NUMERATOR:
Residents who were physically restrained daily (P4c or P4d or P4e = 2) on target assessment

DENOMINATOR:
All residents with a valid target assessment.

COVARIATE(S):
NONE

EXCLUSION(S):
All Medicare Assessments coded in AA8B
Admission Assessment at the Target Assessment
Any P4c, P4d, or P4e has a missing value.

STRATIFICATION:
ADL-Long

74 RSP02 – Percent of Residents who do not have shortness of breath

NUMERATOR:
Residents who do not have shortness of breath (J1l=not checked [Value=0]) AND do not have inability to lie flat due to shortness of breath (J1b=not checked [Value = 0] at Target assessment.

DENOMINATOR:
All residents with a valid target assessment.

COVARIATE(S):
Emphysema/COPD
Age less than 65

EXCLUSION(S):
All Medicare Assessments coded in AA8B
Admission Assessment at the Target Assessment
Residents with non-valid J1l or J1b at the Target assessment.

STRATIFICATION:
PAIN

75 RSPX2 – Percent of Residents who have developed a respiratory infection or have not gotten better

NUMERATOR:
Residents with none of the selected respiratory conditions at prior assessment AND with at least one of the respiratory conditions at the Target assessment (Respiratory Count[t-1]=0 AND respiratory Count[t]>0), OR
Residents with at least one of the respiratory conditions at the Prior assessment AND with the same or a higher count of selected respiratory conditions on the Target assessment than on the Prior assessment
(Respiratory-Count [t-1] > 0 AND Respiratory-Count [t] >= Respiratory-Count [t-1]).
The respiratory conditions included in the count (range 0 to 4) are:
1.Pneumonia (I2e=checked (value 1)).
2.Inability to lie flat due to shortness of breath (J1b=checked (value 1)).
3.Shortness of breath (J1l=checked (value 1)).
4.Recurrent aspirations (J1k=checked (value 1)).

DENOMINATOR:
All residents with a valid target assessment and a valid prior assessment.

COVARIATE(S):
RUG Clincally Complex
Age less than 65
RUG Nursing CMI

EXCLUSION(S):
All Medicare Assessments coded in AA8B
Two Consecutive Chronic Admission Assessments
Residents satisfying any of the following conditions:
Residents with non-valid Respiratory Count at the Target assessment (Respiratory-Count [t] = missing)
Residents with non-valid Respiratory Count at the Prior assessment AND has at least one of the selected respiratory conditions at the Target assessment (Respiratory-Count [t-1] = missing AND Respiratory-Count[t]>0)

STRATIFICATION:
PAIN

76 SOC02 – Percent of Residents engaging in little or no activity

NUMERATOR:
Residents with little or no activity (N2>1) on target assessment.

DENOMINATOR:
All residents with a valid target assessment.

COVARIATE(S):
RUG Nursing CMI
Age less than 65
Pain Scale

EXCLUSION(S):
All Medicare Assessments coded in AA8B
Admission Assessment at the Target Assessment
Residents satisfying any of the following conditions:
The value of N2 is missing on the target assessment.
The resident is comatose (B1 = 1) or comatose status is unknown (B1 = missing) on the target assessment.
The resident has end-stage disease (J5c = checked) or status is unknown (J5c = missing) on the target assessment.

STRATIFICATION:
DRS

77 WAL0X – Percent of Residents who walk as well or better than the previous assessment

NUMERATOR:
Residents with walking in corridor performance scores at the target assessment equal to or less than at the prior assessment (G1d(A)[t]<=G1d(A)[t-1]).

DENOMINATOR:
All residents with a valid target assessment and a valid prior assessment AND one of the following Inclusion Indicators:
1. Capacity to stand (G3a[t-1] = 0, 1, or 2) on the prior assessment or the most recent full assessment.
2. Capacity to walk (G1d(A)[t-1] = 0, 1 or 2) on the prior assessment.

COVARIATE(S):
Full PSI
Age less than 65
RUG Nursing CMI
Cognitive Performance Scale

EXCLUSION(S):
All Medicare Assessments coded in AA8B
Two Consecutive Chronic Admission Assessments
The G1d(A) value is missing on the target assessment [t] or the prior assessment [t-1].
The resident is comatose (B1 = 1) or comatose status is unknown (B1 = missing) on the target assessment.
The resident has end-stage disease (J5c = checked) or status is unknown (J5c = missing) on the target assessment.
The resident is receiving hospice care (P1ao = checked) or hospice status is unknown (P1ao = missing) on the target assessment.

STRATIFICATION:
ADL-Long

78 WALX1 – Percent of Residents who walk as well or better on target assessment as on the prior assessment of their stay.

NUMERATOR:
Residents whose Independence in walking is maintained from the prior assessment to the target assessment: WALK1[t-1]=0 AND WALK1[t]=0 OR
2. Improvement in walking ability is evidenced from the prior assessment to the target assessment (WALK1[t-1] >= WALK1[t])
WALK1 is defined as the sum of G1c(A) and G1d(A) Note: Convert 8's (activity did not occur) to 4's (total dependence) on G1d(A) and G1c(A) for this comparison.

DENOMINATOR:
All residents with a valid target assessment and a valid prior assessment .

COVARIATE(S):
Age less than 65
RUG Nursing CMI

EXCLUSION(S):
All Medicare Assessments coded in AA8B
Two Consecutive Chronic Admission Assessments
Comatose (B1=1) or comatose status unknown (B1 = missing) on the target assessment.
End-stage disease (J5c=checked (value 1)) or end-stage disease status unknown (J5c = missing) on the Target assessment.
The resident is receiving hospice care (P1ao = checked) or hospice status is unknown (P1ao = missing) on the target assessment.
Ventilator dependent (P1al checked (value 1)) or ventilator status is unknown (P1al = missing) on the target assessment.
Quadriplegic (I1z = checked (value 1)) or quadriplegic status is unknown (I1z = missing) on the target assessment.
Paraplegic (I1x = checked (value 1)) or paraplegic status is unknown (I1x = missing) on the target assessment.
Residents with non-valid WALK1 at the target assessment (WALK1[t]=missing)
Residents with non-valid WALK1 at the prior assessment and have some dependence in walking on the target assessment (WALK1[t-1]=missing AND WALK1[t]>0)

STRATIFICATION:
PAIN

79 WGT01 – Percent of Residents who have unexplained weight loss

NUMERATOR:
Residents with weight loss (K3a=1) on target assessment.

DENOMINATOR:
All residents with a valid target assessment.

COVARIATE(S):
Age less than 65

EXCLUSION(S):
All Medicare Assessments coded in AA8B
Admission Assessment at the Target Assessment
Residents satisfying the following condition:
The value of K3a is missing on the target assessment.
Resident has end-stage disease (J5c = checked) or the status of end-stage disease is unknown (J5c = missing) on the target assessment.
The resident is receiving hospice care (P1ao = checked) or hospice status is unknown (P1ao = missing) on the target assessment.
The resident is on a planned weight loss program (K5h = checked) or planned weight loss status in unknown (K5h = missing) on the target assessment.

STRATIFICATION:
CMI

80 Scales

RUG – CMI
This variable is the Resource Utilization Group III, version 5.01, Case-Mix Index resource allocation weight. Each of RUG-III utilization group has a CMI value that reflects the relative cost of caring for a resident in the group, based on nursing resources. RUG-III uses data from MDS assessments to classify continuing care patients into 44 different groups. Groups are defined by use of nursing resources. The original reference for the RUG-III classification system is: Fries, et al. Med Care, 1994; 32:668-85. More information is available on CMS' current use of system at http://cms.hhs.gov/medicaid/reports/rp1201-g.pdf. RUG-III classifies patients into one of seven super-categories: Special Rehabilitation, Extensive Services, Special Care, Clinically Complex, Impaired Cognition, Behavior Problems and Reduced Physical Functions. These-super-categories are further grouped based on activities of daily living (ADL) function. The case-mix index (CMI) for a group in the RUG classification system is the relative weight of resource use in that group compared to average resource use. The CMI weights used in this covariate are the CMI Control Set A01.

RUG Clinically Complex
This covariate is an indicator variable marking whether the resident satisfied the definition for inclusion in the RUG-III Clinically complex group. See Fries et al. (1994) for the original derivation and definition. Residents quality for the clinically complex group if any of the following are true: feeding tube with feeding tube with high parenteral/ enteral intake; comatose and not awake and ADL dependent; septicemia; second or third degree burns; dehydration; hemiplegia/ hemiparesis and an ADL score of ten or more; internal bleeding; pneumonia; end stage disease; chemotherapy; dialysis; physician order changes on 4 or more days and physicians visits on 1 or more day; physician order changes on 2 or more days and physician visits on 7 days; diabetes and injections on 7 days and physician order changes on 2 or more days; transfusions; oxygen therapy; application of dressing to foot and injection on foot or open lesion on foot).

RUG Late Loss ADL
The RUG ADL scale is used to further stratify residents within super-categories, as defined above. It is the sum of four MDS items: bed mobility, transferring, toilet use, and eating. Higher values imply greater ADL impairment. The calculation includes measures of both self-performance and level of support provided by caregivers, using the algorithm:
bed mobility ADL scoretoilet use ADL scoretransfer ADL score = 1 if self-performance<23 if self-performance=24 if self-performance=3|4 & support=25 if self-performance>2 & support>2 , and eating ADL score = 1 if self-performance<22 if self-performacne=23 if self-performance>2 .

The sum has a theoretical range of 4-18. The covariate, as used in the risk-adjustment process, is used as a continuous variable.

RUG - Behavior
This covariate is an indicator variable marking whether the resident satisfied the definition for inclusion in the RUG-III Behavioral problems group. See Fries et al. (1994) for the original derivation and definition. Residents quality for this group if delusions or hallucinations are present, or one of more of the following 4 or more days per week: wandering, verbally abusive behavior, physically abusive behavior, socially inappropriate/ disruptive behavior, resisting care.

RUG Cognitive Impairment
This covariate is an indicator variable marking whether the resident satisfied the definition for inclusion in the RUG-III Behavioral problems group. See Fries et al. (1994) for the original derivation and definition. Residents quality for this group if (Resident must have an ADL index of ten or less and a Cognitive Performance Scale (CPS) of 3 or more (see below for definition of CPS), identifying individuals with moderately pre more severely impaired cognitive functioning.

PSI - Full
This scale, the Morris Frailty Index, is an unweighted sum of MDS assessment items, derived as factors predictive of life expectancy residents. It is coded using data from at least the preceding quarter: both quarterly and full assessment items are used, and the algorithm looks back in the resident's record to the preceding and more remote assessments to identify the appropriate information. A resident's value on the MFI is equal to the number of following indicators present:
Age – 90 or older (A3-year - AA3-year >89)
Cognitive Decision Making – Severely impaired (B4 = 3)
Delirium – Periods of lethargy (B5e = 2)
Ability to Understand – Sometimes/Rarely (C6 = 2,3)
Transfer – Extensive, Total , Did not occur (G1bA = 3,4,8)
Locomotion – Extensive, Total, Did not occur (G1eA = 3,4,8)
Eating – Extensive, Total, Did not occur (G1hA = 3,4,8)
Personal Hygiene – Total, Did not occur (G1jA = 4,8)
Sad Mood, Repetitive Verbalizations – Daily (E1c = 2)
Sad Mood, Something Terrible About to Happen – Daily (E1g = 2)
Acute Episode – Yes (J5b = 1)
Unstable – Yes (J5a = 1)
Change in Care Needs – Deteriorated (Q2 = 2)
End Stage Disease – Yes (J5c = 1)
Bowel – Occasional, Frequent, Incontinent (H1b = 2,3,4)
Weight Loss – Yes (K3a = 1)
Pressure Ulcer – Stages 1 thru 4 (M2a = 1,2,3,4)
Stasis Ulcers – Yes (M2b = 1,2,3,4)

MFI - Subset 1 (Functional)

This covariate is the sum of MFI items that are functional in character that are present: Cognitive Decision Making – Severely impaired (B4 = 3)
Transfer – Extensive, Total , Did not occur (G1bA = 3,4,8)
Locomotion – Extensive, Total, Did not occur (G1eA = 3,4,8)
Eating – Extensive, Total, Did not occur (G1hA = 3,4,8)
Personal Hygiene – Total, Did not occur (G1jA = 4,8)

MFI - Subset 2
This covariate is the sum of MFI items that are not primarily functional in character that are present:
Age – 90 or older (A3-year - AA3-year >89)
Delirium – Periods of lethargy (B5e = 2)
Ability to Understand – Sometimes/Rarely (C6 = 2,3)
Sad Mood, Repetitive Verbalizations – Daily (E1c = 2)
Sad Mood, Something Terrible About to Happen – Daily (E1g = 2)
Acute Episode – Yes (J5b = 1)
Unstable – Yes (J5a = 1)
Change in Care Needs – Deteriorated (Q2 = 2)
End Stage Disease – Yes (J5c = 1)
Bowel – Occasional, Frequent, Incontinent (H1b = 2,3,4)
Weight Loss – Yes (K3a = 1)
Pressure Ulcer – Stages 1 thru 4 (M2a = 1,2,3,4)
Stasis Ulcers – Yes (M2b = 1,2,3,4)

CPS
This covariate, the Cognitive Performance Score, is an index based on MDS data elements that ranks patient's cognitive ability. The original reference is Morris, et al. J Gerontol, 1994; 49:M174-82. The CPS is scored 0-6, with increasing values marking more severe cognitive impairment. Used as a covariate in risk-adjustment models, the CPS is either treated as a categorical variable with the best cognitive functioning level (CPS=0) serving as the reference group or as a set of dummy indicators, each marking membership in one of the CPS levels (omitting CPS=0).
