# Supplementary material for: Adjustment of nursing home quality indicators
Source: BMC Health Serv Res. 2010 Apr 15;10:96. doi: 10.1186/1472-6963-10-96 (PMC2881673; doi:10.1186/1472-6963-10-96)
Supplement: Additional file 3 — Detailed numerical results from the validation analysis. This file contains a table with the detailed results of the validation analysis. [file 1472-6963-10-96-S3.DOC]

##### Jones et al. Adjustment of Nursing Home Quality Indicators

**Additional File 2**

Example of Third Generation Calculation of Nursing Home Quality Indicator

QI Definition: *Proportion of residents who had an unexpected loss of function in some basic daily activities (ADL01)*

***Numerator***: Residents with worsening (increasing item score) in Late-Loss ADL self-performance at target relative to prior assessment. Residents meet the definition of Late-Loss ADL worsening QI if criterion A or B are satisfied:

Criterion A: At least two of the following are true:^1^

1. G1a(A)[t]-G1a(A)[t-1] > 0, or

2. G1b(A)[t]-G1b(A)[t-1] > 0, or

3. G1h(A)[t]-G1h(A)[t-1] > 0, or

4. G1i(A)[t]-G1i(A)[t-1] > 0,

Criterion B: At least one of the following is true:

1. G1a(A)[t]-G1a(A)[t-1] > 1, or

2. G1b(A)[t]-G1b(A)[t-1] > 1, or

3. G1h(A)[t]-G1h(A)[t-1] > 1, or

4. G1i(A)[t]-G1i(A)[t-1] > 1.

Note: Late-Loss ADL items values of 8 are recoded to 4 for evaluation of change.

***Denominator***: All residents with a valid target and a valid prior assessment.

***Covariate***: Client age < 65 years vs. a 65+

***Stratification***: ADL Long form scale (Morris, Fries et al., 1999 J Gerontol A Biol Sci Med Sci 54:M546-M53).

***Example Facility Results***

***Observed QI Scores by Strata***

Strata Number of

Ptile Rank QI Score Residents

ADL-Long Observed Reference Facility Reference

0-19 0.231 0.131 13 53925

20-79 0.135 0.173 37 93501

80+ 0.048 0.099 21 21905

Weighted

Average 0.1268 0.1504

***Expected QI Scores by Strata***^2^

Strata Constant b_1_ ¯_1_ Expected QI^3^

0-19 -1.725 -1.146 0.308 0.111

20-79 -1.535 -0.315 0.027 0.176

80+ -2.171 -0.506 0.000 0.102

***QI Scores by Strata***

Strata QI Score^4^ Computation

0-19 0.265 F^-1^[F(0.131) + F(0.231) - F(0.111)]

20-79 0.133 F^-1^[F(0.173) + F(0.135) - F(0.176)]

80+ 0.046 F^-1^[F(0.099) + F(0.048) - F(0.102)]

***Adjusted QI Score***

*Calculated as weighted average of the strata specific QI scores, using weights from the reference population.*

= 0.164

# Notes

1. G1a(A) refers a specific item on the MDS. Section G, item 1a(A), Self-performance in the activities of daily living area of bed mobility. See the MDS form. [t] refers to the current assessment period (quarter), [t-1] refers to the prior assessment period. Exclusion criteria are not presented in this example.

2. Logistic regression parameters (constant and b1) are obtained from a standard population. In this applied example, the reference population and the standard population for generating the regression weights were the same, and described in the methods section. _1_ is the facility mean on the covariate within strata.

3. The Expected QI score is F^-1^(Constant + b_1_*_1_), where F^-1^ is the inverse logit transformation: F^-1^(z) = 1/1+exp(-1×z), and F(p) is the logit transformation, F(p)=ln(p/(1-p))

4. The Strata specific QI score is computed as F^-1^{F(R) + F(O) - F(E)}, where F is the logit transformation and R is the strata specific proportion with the QI reference population, O is the observed proportion with the QI in the specific strata, and E is the strata specific expected proportion.
