# Supplementary material for: Adjustment of nursing home quality indicators
Source: BMC Health Serv Res. 2010 Apr 15;10:96. doi: 10.1186/1472-6963-10-96 (PMC2881673; doi:10.1186/1472-6963-10-96)
Supplement: Additional file 4 — Figure S2 Summary of validation model analyses. This file contains a figure summarizing the results of the validation analyses of the new third generation quality indicators relative to the Abt/CMS quality indicators. [file 1472-6963-10-96-S4.DOC]

# Additional File 4

**Relative distribution of QI scores in different clinical care settings.**

Each plot illustrates the relative distribution (density, y-axis) of QI scores. Different care settings are illustrated in the same density plot. The x-axis provides marks QI prevalence values, with vertical reference bars marking 0, 20, 40, 60, 80, and 100 percent.

The different care settings are illustrated with the key below.

Abbreviations: NS, Nova Scotia; ON, Ontario; Rehab, Rehabilitation; LTC, Long Term Care; US United States
