# Supplementary material for: Adjustment of nursing home quality indicators
Source: BMC Health Serv Res. 2010 Apr 15;10:96. doi: 10.1186/1472-6963-10-96 (PMC2881673; doi:10.1186/1472-6963-10-96)
Supplement: Additional file 5 — Table S1. Characteristics of Residents and Facilities. This file contains a table describing the characteristics of the residents and facilities used in the analysis. [file 1472-6963-10-96-S5.DOC]

# Additional File 5

GEN2 is the autocorrelation (quarter-to-quarter) of second generation adjusted QIs. Missing values indicate the QI did not exist as a second generation QI. GEN3 is the autocorrelation of third generation adjusted QIs.
