# Supplementary material for: Adjustment of nursing home quality indicators
Source: BMC Health Serv Res. 2010 Apr 15;10:96. doi: 10.1186/1472-6963-10-96 (PMC2881673; doi:10.1186/1472-6963-10-96)
Supplement: Additional file 6 — Detailed numerical results from the reliability analysis. This file contains a table with the results of the reliability analysis (quarter to quarter autocorrelation) for the second and third generation quality indicators. [file 1472-6963-10-96-S6.DOC]

# Additional file 6

# Validation Runs – Numerical Results

Notes: All Original QI Validation Elements (centered, missing values regression imputed) Using first 10 principal components from among all preventive, responsive elements. N describes the number of facilities, Validity is the assignment of level of validity using rules developed in the Mega-QI project. Missing values for second generation QIs indicates that the QI was not part of the second generation QI set. N describes the number of facilities, MRp describes the preventive multiple correlation, MRr describes the preventive multiple correlation, and MRt describes the total (preventive and responsive) multiple correlation. Validity is the assignment of level of validity using rules developed in the Mega-QI project.
