# Supplementary material for: Adjustment of nursing home quality indicators
Source: BMC Health Serv Res. 2010 Apr 15;10:96. doi: 10.1186/1472-6963-10-96 (PMC2881673; doi:10.1186/1472-6963-10-96)
Supplement: Additional file 7 — Figure S1 Scattergraph of autocorrelation coefficients for third and second generation quality indicators. This file contains a figure illustrating the correlation of autocorrelation coefficients for third and second generation quality indicators. [file 1472-6963-10-96-S7.DOC]

**Figure 1. Autocorrelation (quarter to quarter correlation) of Quality Indicators scored using new risk adjustment methodology (third generation) versus CMS/Abt Associates methodology (second generation).**
