# Supplementary material for: Adjustment of nursing home quality indicators
Source: BMC Health Serv Res. 2010 Apr 15;10:96. doi: 10.1186/1472-6963-10-96 (PMC2881673; doi:10.1186/1472-6963-10-96)
Supplement: Additional file 8 — Density plots of each quality indicator. This document contains a density plot for each quality indicator for each major care sector. [file 1472-6963-10-96-S8.DOC]

**Figure 2. Validity analysis of Quality Indicators computed using Abt Associates/CMS risk adjustment methodology (second generation) and new risk adjustment method (third generation).**

Note: QI abbreviations match those listed in Additional File 2
